# Touchscreen-Based Cognitive Training Alters Functional Connectivity Patterns in Aged But Not Young Male Rats

**DOI:** 10.1523/ENEURO.0329-22.2023

**Published:** 2023-02-23

**Authors:** Leslie S. Gaynor, Meena Ravi, Sabrina Zequeira, Andreina M. Hampton, Wonn S. Pyon, Samantha Smith, Luis M. Colon-Perez, Marjory Pompilus, Jennifer L. Bizon, Andrew P. Maurer, Marcelo Febo, Sara N. Burke

**Affiliations:** 1 Memory and Aging Center, Department of Neurology, University of California, San Francisco, CA 94158; 2 Department of Neuroscience, University of Florida, Gainesville, FL 32610; 3 Department of Psychiatry, University of Florida, Gainesville, FL 32610; 4 McKnight Brain Institute and College of Medicine, University of Florida, Gainesville, FL 32610; 5 Department of Pharmacology and Neuroscience, University of North Texas Health Science Center, Fort Worth, TX 76107

**Keywords:** associative memory, cognitive aging, cognitive training, functional connectivity, graph theory, medial temporal lobe

## Abstract

Age-related cognitive decline is related to cellular and systems-level disruptions across multiple brain regions. Because age-related cellular changes within different structures do not show the same patterns of dysfunction, interventions aimed at optimizing function of large-scale brain networks may show greater efficacy at improving cognitive outcomes in older adults than traditional pharmacotherapies. The current study aimed to leverage a preclinical rat model of aging to determine whether cognitive training in young and aged male rats with a computerized paired-associates learning (PAL) task resulted in changes in global resting-state functional connectivity. Moreover, seed-based functional connectivity was used to examine resting state connectivity of cortical areas involved in object-location associative memory and vulnerable in old age, namely the medial temporal lobe (MTL; hippocampal cortex and perirhinal cortex), retrosplenial cortex (RSC), and frontal cortical areas (prelimbic and infralimbic cortices). There was an age-related increase in global functional connectivity between baseline and post-training resting state scans in aged, cognitively trained rats. This change in connectivity following cognitive training was not observed in young animals, or rats that traversed a track for a reward between scan sessions. Relatedly, an increase in connectivity between perirhinal and prelimbic cortices, as well as reduced reciprocal connectivity within the RSC, was found in aged rats that underwent cognitive training, but not the other groups. Subnetwork activation was associated with task performance across age groups. Greater global functional connectivity and connectivity between task-relevant brain regions may elucidate compensatory mechanisms that can be engaged by cognitive training.

## Significance Statement

Cognitive impairments in advanced age have been linked to reduced resting state functional connectivity in humans and rats. Resting state functional connectivity is typically examined at a single time point. Thus, little is known regarding the robust versus adaptive properties of these networks in old age. The current study reports longitudinal changes in global connectivity as well as connectivity involving the medial temporal, frontal, and retrosplenial (RSC) cortices in aged rats that were trained on a rodent touchscreen version of the paired-associates learning (PAL) task. This work is significant because it shows that the functional organization of brain networks in aged rats is adaptive, and this could be leveraged to design behavioral therapies for improving cognitive function in old age.

## Introduction

Advanced age is associated with neurobiological alterations within the frontal cortices (Barense et al., 2002; Cabeza et al., 2004; Bañuelos et al., 2014) and medial temporal lobe (MTL; Rosenzweig and Barnes, 2003; Burke et al., 2018). Because specific cellular changes within distinct subregions of the frontal-MTL network are not uniform, targeted interventions that restore function in one brain region may neglect or exacerbate dysfunction in another, hindering the restoration of normal cognition. Thus, interventions aimed at optimizing network function may be more effective at improving cognitive outcomes in older adults. The development of therapeutic strategies for enhancing network function, however, is impeded by a lack of understanding of how neurobiological changes at the cellular level, identified from invasive studies in animals, influence the organization of functional brain networks examined in humans. Inferring resting state functional connectivity from correlated or anti-correlated regional BOLD fluctuations across the rodent lifespan (Ash et al., 2016; Colon-Perez et al., 2019; Liang et al., 2020) can facilitate linking cellular changes derived from invasive studies to large-scale brain network organization (Colon-Perez et al., 2019; Xu et al., 2022) across species.

Tests of associative learning and memory are potentially valuable for understanding the link between cellular dysfunction and functional connectome organization in the context of cognitive aging, as these behaviors are known to engage distributed neural networks (Glisky et al., 1995; Moscovitch and Winocur, 1995; Buckner, 2004). In animal models, recognition of object-place associations requires functional connectivity between frontal and MTL regions, including the hippocampus and perirhinal cortex (Barker and Warburton, 2015). Moreover, acquisition of object-place associations requires frontal and MTL regions (J. Kim et al., 2011; Hernandez et al., 2017). Both the acquisition of object-place associations (Hernandez et al., 2015, 2019) and functional connectivity between frontal-MTL networks that support this behavior are known to be disrupted in advanced age (Hernandez et al., 2018, 2020b). Frontal-MTL connectivity is also mediated by the retrosplenial cortex (RSC; Kaboodvand et al., 2018), which is a hub of the default mode network in both humans (Kaboodvand et al., 2018) and rats (Hsu et al., 2016). Relatedly, age-related cognitive deficits are associated with lower RSC-hippocampus functional connectivity in aged humans (Ziontz et al., 2021). In rats, lower functional connectivity between the RSC and sensorimotor cortex, posterior parietal/secondary visual cortex, and dorsal auditory/temporal association cortex with age is associated with worse performance on the Morris watermaze (Ash et al., 2016; Nasrallah et al., 2016). Tasks used for assessing associative learning and memory in rodents and humans, however, have traditionally used cognitive testing procedures that are not comparable. For example, rodents are often tested in large mazes with three-dimensional stimuli while humans are tested on computers with images.

Operant touchscreen cognitive testing in rodents allows preclinical researchers to develop, expand, and validate rodent models (Talpos et al., 2009; Bartko et al., 2011; C.H. Kim et al., 2015; J. Kim et al., 2016) with behavioral tasks that are more homologous to those performed in clinical human studies (Horner et al., 2013). The paired-associates learning (PAL) task is a computerized task that has been adapted from humans [human task developed as part of the Cambridge Neuropsychological Test Automated Battery (CANTAB) battery] for testing visuospatial object-location associative learning and memory in rodents. PAL task performance declines with age in both humans (Robbins et al., 1994; Lee et al., 2013) as well as rats (Smith et al., 2022), and is sensitive to detecting impairment in older adults with early-stage Alzheimer’s disease (Egerházi et al., 2007). While PAL is potentially a useful translational tool for understanding neurobiological aging, it is not known the extent to which performance on this task is related to brain network organization in rodents. Thus, the current study examined PAL acquisition in young and aged rats to determine the extent that cognitive training and performance on this task was associated with changes in global resting state functional connectivity as well as functional connectivity between the MTL (perirhinal cortex and hippocampus), RSC, and frontal cortical areas (infralimbic and prelimbic cortices).

## Materials and Methods

### Subjects and handling

A total of 17 young adult (four months old) and 20 aged (22–28 months old) male Fischer 344 × Brown Norway F1 hybrid rats (NIA colony) were used for this study. Twelve young and 12 aged rats underwent cognitive training. An additional five young and eight aged rats were trained to traverse a track for a food reward but did not undergo cognitive training. This group was also scanned longitudinally at a similar interval as the cognitively trained animals, was similarly food restricted, received palatable food rewards for traversing a track, and was considered the activity-matched control group. Because of the lack of availability of female rats of this strain at the time of these experiments, sex as a biological variable could not be considered.

Rats were single-housed in standard Plexiglas cages and maintained on a 12-h reverse light/dark cycle (lights off at 8 A.M.). All rodent handling, feeding, and behavior was conducted during the dark phase, 5–7 d per week at approximately the same time each day. Upon arrival, rats were given one week of acclimatization to the facility. They were then placed on food restriction to induce appetitive motivation before behavioral testing. The diet consisted of moist chow (standard rat maintenance diet, Purina, and water 1:1 ratio). Throughout training and testing, rats were weighed daily to ensure they maintained a target weight between 80–85% of their normal baseline weight (i.e., the weight at which they have a body condition score, or BCS, of 3). Rats also underwent weekly health screens to ensure their BCS did not drop below 2.5 and that they did not acquire tumors or other physical impairments. BCS for each rat were assigned by assessing palpable fat deposits over the lumbar vertebrae and pelvic bones (Ullman-Culleré and Foltz, 1999; Hickman and Swan, 2010). Water was provided *ad libitum*. All experimental procedures were performed in accordance with National Institutes of Health guidelines and were approved by Institutional Animal Care and Use Committees at the University of Florida.

### Cognitive training apparatus

All training and experimental procedures were conducted in eight rodent touchscreen operant chambers (Bussey-Saksida Rat Touchscreen Chamber, Lafayette Instruments) and ABET II software for touchscreens was used for experimental control and data recording (Lafayette Instruments). Each touchscreen chamber (21.6 × 17.8 × 12.7 cm) was housed within a sound-attenuating box (Med Associates Inc.) and consisted of a touchscreen monitor (15.0-inch, screen resolution 1024 × 768), fan (providing ventilation and white noise), stainless steel grid floor, tone generator, house light (LED), magazine unit (with light and infrared beam to detect entries), and reward dispenser. Liquid reward (Ensure Light Vanilla) was used. The inner chambers were composed of three black plastic walls, trapezoidal in shape to help guide the focus of the animal toward the touchscreen and reward delivery area. A black Perspex “mask” (h 38 × 28 cm) was placed over the touchscreen monitor with three response windows (h 15 × 6 cm) cut into it. Response windows were 1.5 cm apart, while the outer response windows were 4.5 cm from the edge of the mask. Attached at a 90° angle 16 cm above the stainless-steel grid floor was a spring-hinged “shelf” (d 6 cm, w 20.5 cm). The positioning of the shelf required rats to stop and rear up toward the screen to explicitly facilitate attention toward touchscreen stimuli. All touchscreen procedures including presentation of stimuli and data collection were performed using the ABET II Touchscreen software (Lafayette Instrument Co) and WhiskerServer interface (Cambridge University Technical Services Ltd.) installed on a Windows PC.

### Shaping and behavioral procedures

Rats were initially shaped to use the touchscreen through five incremental training stages: Magazine, Any Touch, Must Touch, Must Initiate, and Punish Incorrect. Rats were first introduced to the touchscreen chamber through magazine training, in which nose poking the magazine opening elicited 50 μl of the liquid Ensure reward to be dispensed. Rats were trained to associate the liquid reward with a tone until they reliably completed 100 trials in 45 min. The screen remained inactive during this stage. Criterion for all subsequent stages of shaping required rats to complete 80 trials in 45 min.

During the Any Touch stage, rats were shaped to engage with the touchscreen and offered a food reward for touching any white square presented on the stimulus windows of the screen. Upon encountering difficulties getting aged rats to consistently engage with the touchscreen during the Any Touch stage, a modified shaping paradigm was adapted to include three additional stages which took place after Magazine training: Any Touch Fullscreen, Any Touch Progressive, and Any Touch Shelf. These stages were developed to gradually direct the rat’s attention to the portion of the screen where the stimuli would later be presented, and to introduce the rat to the shelf to assist with rearing to approach the presented stimuli. These stages are discussed in detail in a previous paper (Smith et al., 2022).

Upon completion of Any Touch, rats progressed to the Must Touch stage. Unlike the previous stage of shaping, only one white square was illuminated per trial. The rat was required to select the white square to receive a reward. Touches to the blank panels were not rewarded in this stage of shaping. To prevent the development of a side bias, the location of the illuminated panel was pseudo-randomized. The next stage in shaping, Must Initiate, added a required nose poke of the feeder to initiate a new trial. The final stage of shaping, Punish Incorrect, introduced a time out period for nose poking a blank panel in which the house light was illuminated for 10 s before a new trial could be initiated.

### Paired-associates learning (PAL)

**Figure 1.**
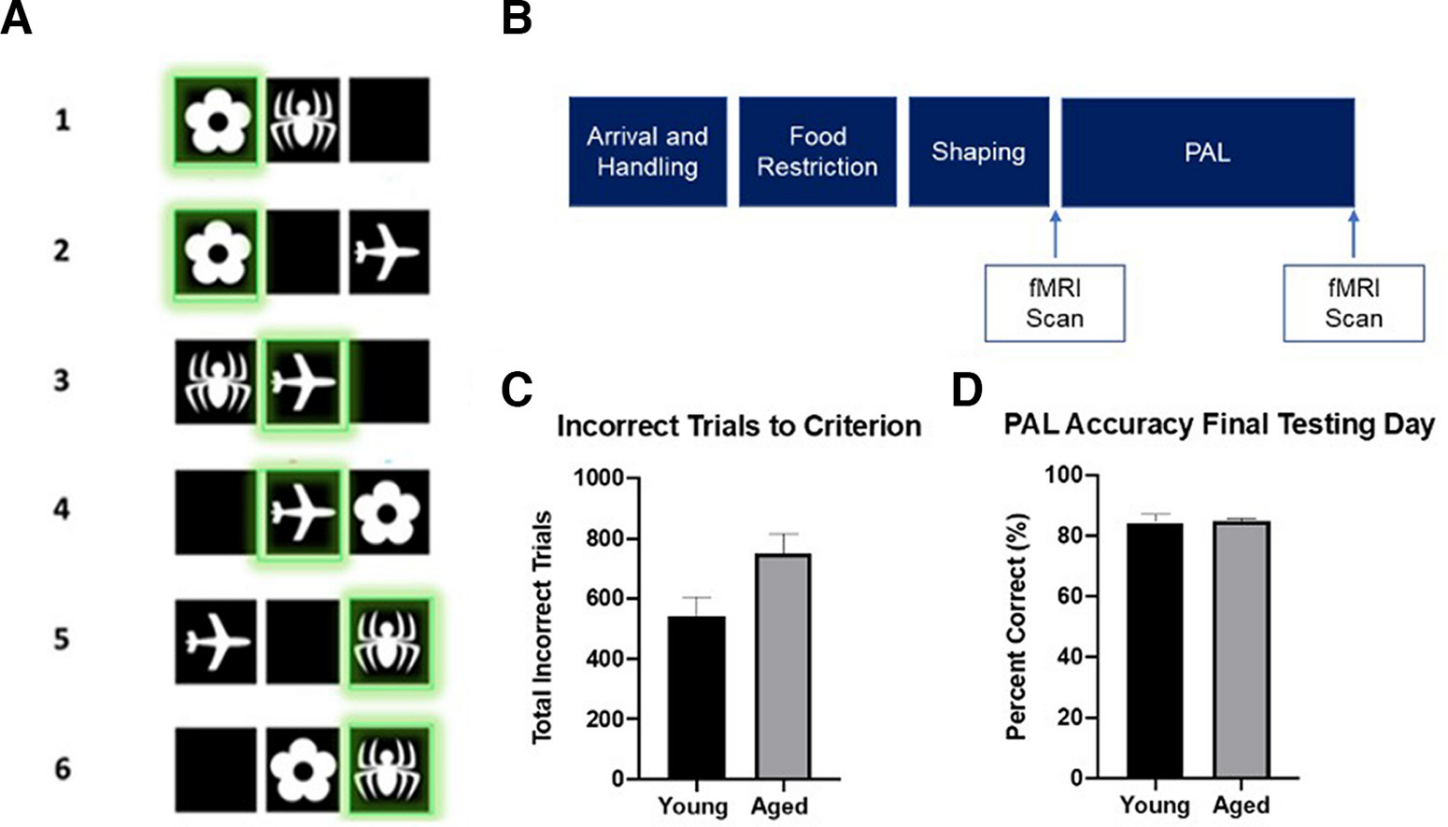
PAL touchscreen task shaping and training timeline and behavioral performance across age groups. ***A***, PAL stimuli and the six different trial types. Green square indicates the correct stimulus. ***B***, Timeline depicting shaping and training protocol for PAL task. ***C***, Bar graph depicting total incorrect trials to PAL criterion across age groups. Independent-samples *t* test revealed that aged rats made significantly more errors compared with young rats (*t*_(20)_ = 2.34, *p* = 0.03). ***D***, Bar graph depicting PAL percent accuracy on the final testing day across age groups. There was not a significant difference in PAL percent accuracy between age groups (*t*_(13.33)_ = −0.18, *p* = 0.86). Error bars indicate ±1 standard error of the mean (SEM).

The PAL task consists of presentation of three possible “object” stimuli (O1, O2, O3) in three possible locations (L1, L2, L3; Fig. 1*A*). Each of the three stimuli were assigned to a unique location in one of the three panels, which was considered its “correct” location. During each trial, two stimuli were presented simultaneously in two of the three possible locations. One stimulus was presented in its correct location (S+), whereas the other stimulus was presented in an incorrect location (S−). There were six possible trial types: (1) S+ = L1O1, S− = L2O3; (2) S+ = L1O1, S− = L3O2; (3) S+ = L2O2, S− = L1O3; (4) S+ = L2O2, S− = L3O1; (5) S+ = L3O3, S− = L1O2; (6) S+ = L3O3, S− = L2O1, as shown in Figure 1*A*. Trials were pseudo-randomized to ensure that no specific trial type was presented consecutively more than three times. During the first several days of PAL testing, rats completed the PAL task with correction trials to gain familiarity with the behavioral stimuli before testing. During the PAL acquisition phase, responses to the incorrect stimulus (S−) were ignored and the rat was able to continue responding until the target stimulus was selected (S+), which was then treated as a correct response and rewarded. Selection of the correct stimulus was followed by an intertrial interval (ITI) of 10 s. After rats reached criterion of two consecutive days of 90 completed trials or 180 trials total of PAL acquisition with correction trials, they proceeded to the regular PAL testing, in which selection of an incorrect object-location pairing (S−) was followed by no food reward and a 10-s time out during which the stimuli were removed from the screen and the house light turned on. Testing continued until a criterion of 80% correct and 80 trials across 3 d was met.

### Activity-matched controls

A group of control rats that did not undergo cognitive training with PAL were also examined. In this second group of rats, animals were first food restricted as described above. They were then shaped to traverse a circular track (outer diameter: 115 cm, inner diameter: 88 cm) for Froot Loop reward for ∼30–45 min each day to match the duration of PAL testing. Rats completed 32 laps around the circular track per day. Walking on the track occurred daily at approximately the same time. For control rats, imaging sessions were completed on days 11 and 27 of track running.

### Functional magnetic resonance imaging

Each rat underwent two fMRI sessions. For rats that completed cognitive training, the first scan was acquired after all shaping procedures had been completed, following punish incorrect and before the PAL acquisition phase. The second imaging session was acquired after each rat met criteria on the PAL task. Because young rats reached criterion performance in fewer days than did the aged rats (Smith et al., 2022), they would continue testing on the touchscreens until they could be scanned with a group of aged rats and the MRI became available. On average there were 69.9 d between scans for the young rats and 74.9 d between scans for the aged rats, which was not statistically different (*t*_(20)_ = 0.68, *p* = 0.50). During both sessions, rats were subject to similar food restriction and handling procedures.

At both scanning sessions, rats were imaged under isoflurane (1.5%) sedation (delivered in 70% N_2_/30% O_2_ at 0.1 l/min via inhalation). Despite vasodilation and suppression of neural activity, inhalation anesthetic agents, such as isoflurane, are preferred over intravenous injectable anesthetic agents in longitudinal fMRI studies because of fast recovery, lower mortality, and easier control of sedative levels in the blood (Masamoto et al., 2007; Williams et al., 2010). BOLD activation patterns have been successfully measured with low levels of anesthetics in animals (Masamoto et al., 2007; Kannurpatti et al., 2008; T. Kim et al., 2010; Williams et al., 2010) and humans (Riehl et al., 2018). Longitudinal rsfMRI studies have also been completed in which functional connectivity changes were measured in sedated rodents after cognitive training with a learning and memory task (Ash et al., 2016; Nasrallah et al., 2016; Colon-Perez et al., 2019). For the present study, isoflurane levels were kept below 2% during functional imaging to limit dose-dependent vasodilation. Thus, sedation was initially induced with 3–4% isoflurane, but then lowered and fixed at 1.5% for the duration of the experiment. Spontaneous breathing was monitored throughout MRI acquisition and maintained between 30–50 average breaths per minute (SA Instruments). A warm water recirculation system was used to maintain body temperature at 37–38°C.

Resting state fMRI data were collected with a 4.7/33-cm horizontal Tesla magnet (Magnex Scientific) using an 11.5-cm diameter gradient insert (Resonance Research, Billerca, MA; 670 mT/m maximum gradient strength at 300 A and 120-μs rise time) and controlled using VnmrJ 3.1 console software (Agilent). A 38-mm quadrature transmit/receive radio frequency (RF) coil tuned to 200.6 MHz^1^H resonance was used for B_1_ field excitation and RF signal detection (aimri; LLC). Images were acquired using a two-shot spin echo echoplanar imaging (or EPI) sequence with the following parameters: TR/TE = 1000/50 ms and 300 repetitions for a total acquisition time of ∼10 min (an image was acquired every 2 s), FOV = 32.5 × 32.5 mm^2^, 12 slices 1.5 mm thick, and data matrix = 64 × 64. Anatomical scans for imaging overlay and reference-to-atlas registration were collected using a fast spin echo sequence with the following parameters: TR/TE_eff_ = 2000/45 ms, RARE factor = 8, number of averages = 10, data matrix = 256 × 256, and the same space as the EPI scan.

### Image processing

Brain masks were manually drawn over high-resolution anatomic scans using segmentation tools in (www.itksnap.org). Brain masks were used to crop images and remove nonbrain voxels. FMRIB Software Library linear registration program flirt (Jenkinson et al., 2002) was used to align cropped brain images with rat brain template using previously published parameters (Colon-Perez et al., 2016). Registration matrices were saved and then used to transform functional datasets into atlas space for preprocessing and analysis. Analysis of Functional NeuroImages (Cox, 1996) was used to correct image displacements, slice timing delays, and remove time series spikes. Other preprocessing steps included linear and quadratic detrending, spatial blurring, and intensity normalization. Head motion parameters and cerebroventricular and white matter signals were removed from all datasets. Brain signals containing cardiac and respiratory frequencies were removed with a voxelwise temporal bandpass filter (between 0.01 and 0.1 Hz).

Time-series fMRI signals were extracted from regions of interest (ROI) determined by atlas-guided seed location (150 total structures, 75 per hemisphere). This segmentation atlas is based on the Paxinos and Watson (Paxinos and Watson, 1997) rat brain atlas and was originally developed by Ekam imaging (Craig Ferris, Northeastern University, Boston, MA; Yee et al., 2015). This atlas has been used in several publications (Colon-Perez et al., 2018, 2019). Signals were averaged from voxels in each ROI (Colon-Perez et al., 2016). Voxel-wise cross-correlations were conducted to create correlation coefficient (Pearson’s R) maps between all 150 structures. Correlation maps were subjected to voxelwise z-transformation. The first nine images in each functional time series were not used in the cross-correlation step. The two correlation maps were averaged per subject to generate a single correlation map used for statistical mapping. AFNI’s 3dClustSim program was used to determine adequate cluster size for a given uncorrected *p*-value. The resultant voxel cluster size (*p* ≤ 0.05) was used to control the level of false positive rates in the final composite statistical maps.

### Network analysis

Brain connectivity networks were analyzed using the Brain Connectivity Toolbox for MATLAB (Rubinov and Sporns, 2010), as in previous publications (Colon-Perez et al., 2018; Orsini et al., 2018). First, symmetrical connectivity graphs (11,175 matrix entries) were organized in MATLAB. Then, *z* score thresholding was used to create matrices of equal densities, such that only *z* score values ≥15 were included. Matrix *z* score values were normalized by the highest *z* score value such that all matrices had edge weight values ranging from 0 to 1. *Z* score values were used to create values of node strength, which is the sum of edge weights, node degree, which is the number of edges connected to a node, and clustering coefficient, which is the degree to which nodes cluster together in groups. Three-dimensional whole-brain and modular brain networks were visualized using BrainNet (Xia et al., 2013). Networks were generated using undirected edge weights (E_undir_ ≥ 0.3), with node size and color scaled by node strength and edge weights scaled by *z* scores.

### Statistical analyses

Analyses were performed with the Statistical Package for the Social Sciences (SPSS IBM Inc, Armonk, NY) v25 for Windows; *p*-values < 0.05 were considered statistically significant. PAL task behavioral data were examined using repeated measures ANOVA with the between-subjects factors of age (young, aged) and within-subjects factor of scan session (scan 1, scan 2). Brain network graphs and whole-brain matrices were evaluated to determine whether changes in whole-brain connectivity across scan sessions differed by age. Global connectivity metrics, including global strength (the sum of edge weights), clustering coefficient (the degree to which nodes cluster together in groups), and modularity (the degree to which the network may be subdivided into clearly delineated groups or communities) were examined across groups.

Node degree and strength were also observed across age and scan session, and a repeated measures ANOVA was performed to determine whether the number of high degree (>10) and high strength (>15) nodes differed by age and scan session within the Cognitive Training and Walking conditions. The Cognitive Training and Walking conditions were not directly compared because of data being acquired at different time points and differences in the length of time between scans across conditions.

To assess whether the connectivity of specific networks increased following cognitive training, the 20 highest strength nodes and highest degree nodes (10 per hemisphere, given lack of evidence of laterality) for aged rats at scan session 2 were selected and a repeated measures ANOVA was used to evaluate modularity of each of these networks across scan session in both young and aged rats. These analyses were repeated for age-matched controls. Repeated measures ANOVAs were also performed to assess group mean differences in seed-based connectivity and clustering coefficients of brain structures of interest across scan session and age. Brain regions were examined bilaterally because of insufficient evidence to defend lateralization of age-related changes in rodent models of aging.

To assess whether brain changes were related to task performance, stepwise linear regression was performed to determine whether there were notable relationships between PAL task performance (specifically, PAL percent correct at day 15) and change in functional variables for which there were significant differences between age groups and across scan sessions.

## Results

### Cognitive training

The age-associated impairment on PAL performance is described in detail elsewhere (Smith et al., 2022). Briefly, at day 15 of testing, the young rats were significantly more accurate than the aged animals at selecting the correct image (*t*_(20)_ = 2.61, *p* = 0.01, *d *=* *1.04). Moreover, aged rats made significantly more errors compared with young before reaching PAL criterion (*t*_(20)_ = 2.34, *p* = 0.03, *d *=* *1.0; Fig. 1*C*). On the final testing day, before the second scanning session, there was not a significant difference between age groups in terms of performance accuracy (*t*_(20)_ = −0.18, *p* = 0.86; Fig. 1*D*). This observation indicates that aged rats were able to acquire stimulus-location associations after a greater number of trials compared with young rats.

### Whole-brain connectivity

**Figure 2.**
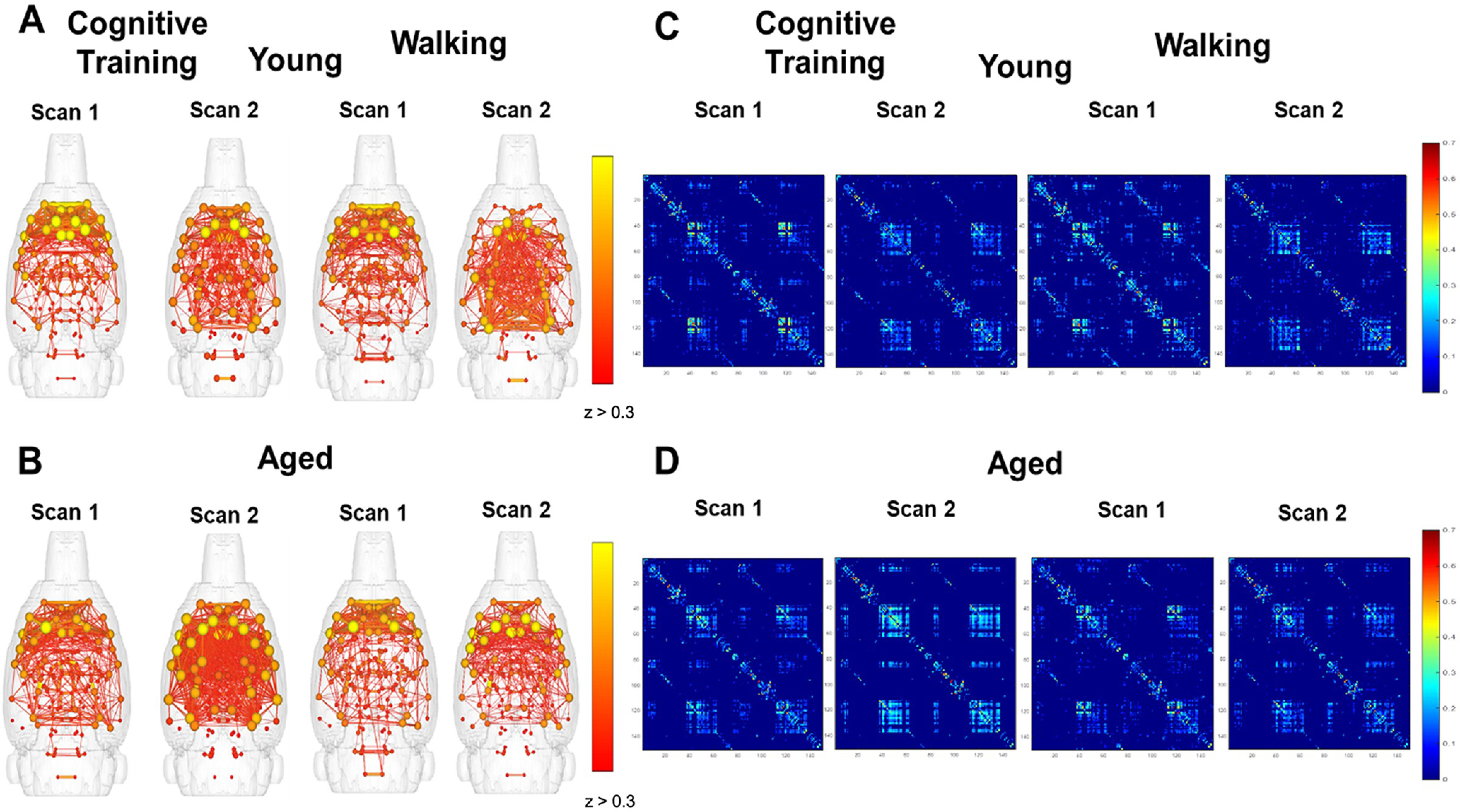
Effects of cognitive training or walking on global functional connectivity in young and aged rats. ***A, B***, Whole-brain connectivity maps by scan session and age group for rats that underwent cognitive training versus walking around a track for reward. The brain network maps represent connected nodes, the spheres indicating the strength of node connectivity to the whole network. Edges represent significant correlations in the BOLD fluctuations between two nodes and are depicted by lines when the correlation is *z *>* *0.3. ***C***, ***D***, Whole-brain correlation matrices for the 150 segmented brain regions by scan session and age group for the Cognitive Training or Walking conditions.

Brain network graphs and whole-brain matrices were evaluated to examine for potential changes in whole-brain connectivity patterns across scan sessions for young and aged rats that underwent Cognitive Training versus the Walking condition. To examine whether Cognitive Training or Walking altered the global connectome, brain network maps were generated in which connected modules with edges *z* ≥ 0.3 were displayed. Figure 2*A*,*B* shows the connectivity maps for young (top) and aged (bottom) rats that underwent PAL Cognitive Training (left) or completed the Walking condition (right), which had a limited cognitive load compared with PAL. Node strength is represented by the spheres, with yellow indicating greater connectivity of the node to the entire network. Significant edges between nodes are indicated with lines. Qualitatively, these maps suggest that there was a greater number of significantly correlated nodes at scan 2 relative to scan 1 in aged rats that underwent PAL Cognitive Training, but not young rats. The comparable plots for the young and aged rats from the Walking condition indicate that the pattern of increased connectivity in aged animals is only present following cognitive training, but not walking. Figure 2*C*,*D* shows the connectivity matrices for the 150 segmented brain regions, which also depicts an enhanced connectivity between some brain regions in aged rats following cognitive training that is not evident following the Walking condition.

**Figure 3.**
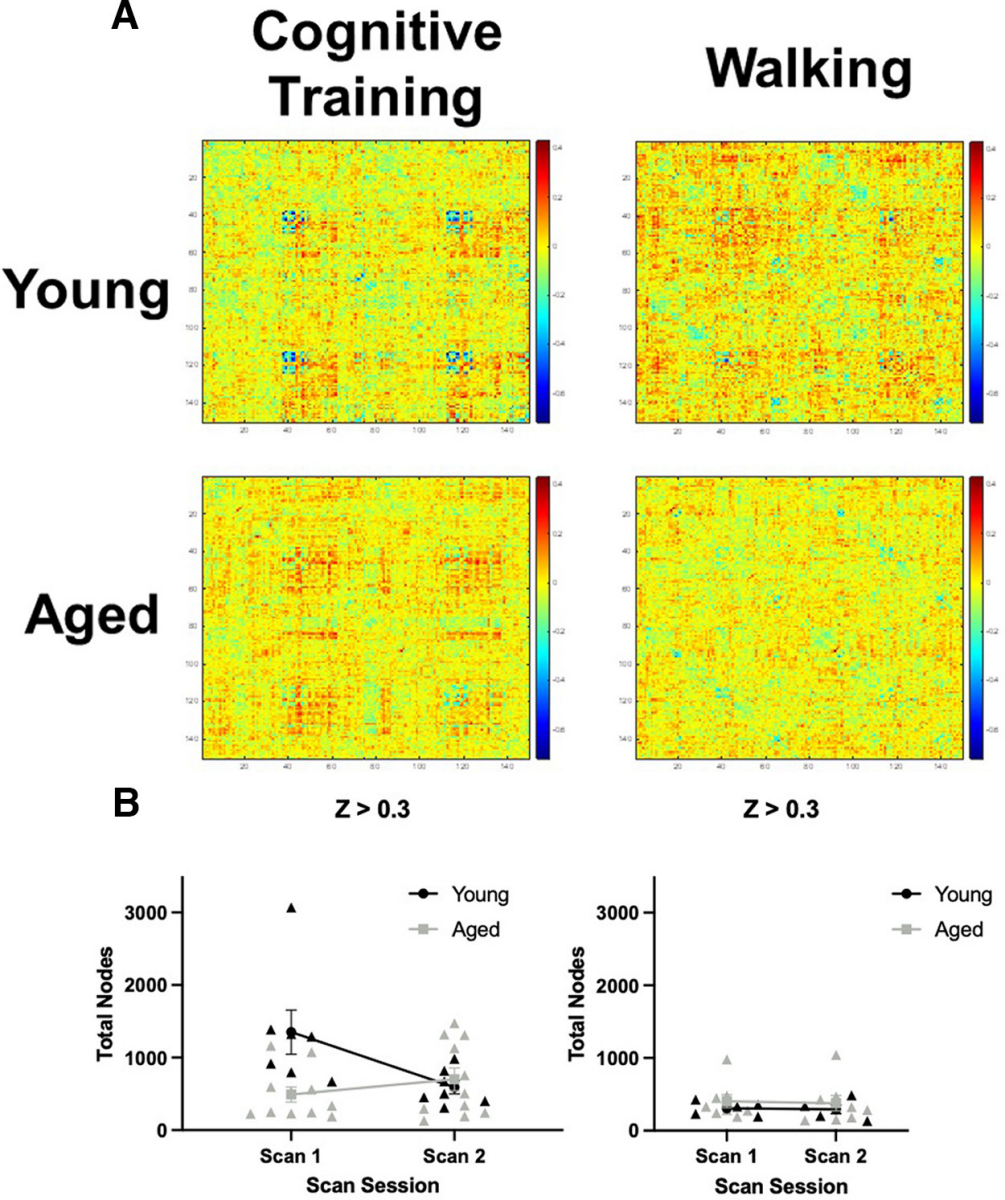
Change in global functional connectivity across scan sessions following Cognitive Training or Walking. ***A***, The difference between scan 1 and scan 2 whole-brain correlation matrices from young (top) and aged (bottom) rats for the Cognitive Training (Young: *N* = 7; Aged: *N* = 11) versus Walking (Young: *N* = 5; Aged: *N* = 8) conditions. ***B***, Line graphs representing the number of significant edges (*z* ≥ 0.3) across scan sessions by age for the Cognitive Training (left) or Walking (right) conditions. There was a significant effect of age (*F*_(1,16)_ = 5.23, *p* = 0.04, η^2^ = 0.25) and a significant interaction effect of scan session and age (*F*_(1,16)_ = 7.25, *p* = 0.02, η^2^ = 0.31), indicating that aged rats had fewer edges compared with young rats at scan 1, and that while the number of these edges increased at scan session 2 for aged rats, the number of edges decreased for young rats. In the Walking condition, there were no significant effects of age or scan session on edge number. Error bars are ± 1 SEM.

To better understand functional connectivity changes occurring across scan sessions, the correlation matrices from scan 1 were subtracted from scan 2 (Fig. 3*A*). Warmer colors indicate a greater enhancement of correlations in scan 2 relative to scan 1. These difference matrices suggest that, in addition to the aged rats in the Cognitive Training condition displaying enhanced functional connectivity at scan 2, there were also brain regions that were more weakly correlated in young rats at scan 2 (indicated by blue) that show little to no change across scans in aged rats. Notably, there were less areas of positive change for aged rats in the Walking condition that did not receive cognitive training.

Given these observations, we evaluated differences in the number of edges with z ≥ 0.3 across scan sessions by age group (Fig. 3*B*). For the Cognitive Training condition, repeated-measures ANOVA did not reveal a significant within-subjects effect of scan session on the number of edges *z* ≥ 0.3 (*F*_(1,16)_ = 2.34, *p* = 0.15). However, there was a significant effect of age (*F*_(1,16)_ = 5.23, *p* = 0.04, η^2^ = 0.25) and a significant interaction effect of scan session and age (*F*_(1,16)_ = 7.25, *p* = 0.02, η^2^ = 0.31). These results indicate that aged rats had fewer edges compared with young rats at scan 1, and that while the number of these edges increased at scan session 2 for aged rats, the number of edges decreased for young rats. In the Walking condition, there was not a significant effect of scan session (*F*_(1,11)_ = 0.04, *p* = 0.85), age (*F*_(1,11)_ = 1.50, *p* = 0.25), or a significant interaction of scan session and age (*F*_(1,11)_ = 0.002, *p* = 0.96) on edges *z* ≥ 0.3. Of note, there was a difference at scan 1 in the number of edges with *z* ≥ 0.3 between young rats in the Cognitive Training and Walking conditions. This difference was not present at scan 2 given the reduced number of edges with *z* ≥ 0.3 at scan 2 in young rats in the Cognitive Training condition.

**Figure 4.**
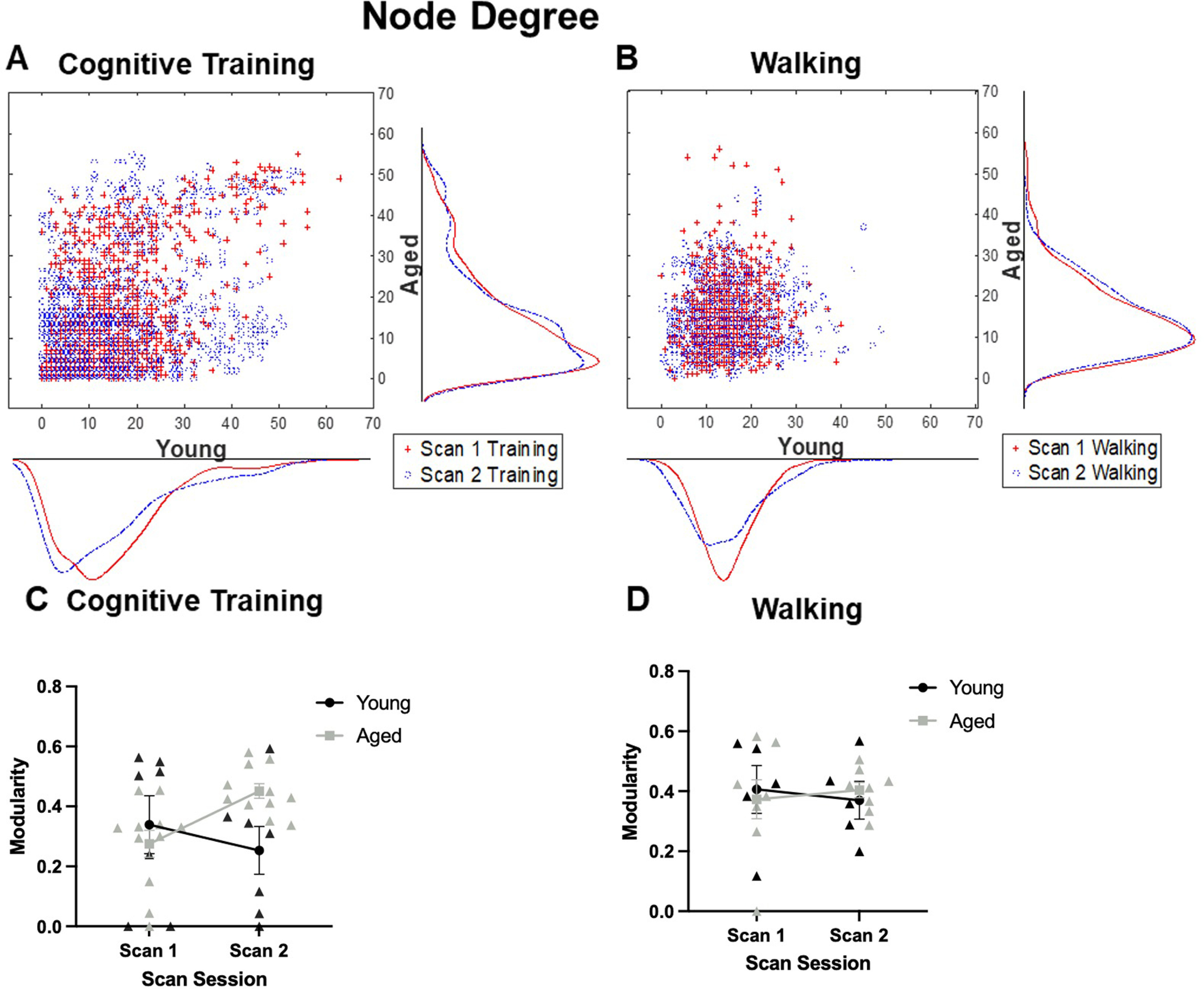
Node degree and modularity of high degree nodes. ***A***, Scatter histograms representing node degree in young and aged rats across scan sessions relative to Cognitive Training (Young: *N* = 7; Aged: *N* = 11) or (***B***) Walking (Young: *N* = 5; Aged: *N* = 8). There was not a significant main effect of age (Cognitive Training: *F*_(1,16)_ = 0.70, *p* = 0.42; Walking: *F*_(1,11)_ = 0.03, *p* = 0.86) or scan session (Cognitive Training: *F*_(1,16)_ = 0.40, *p* = 0.54; Walking: *F*_(1,11)_ = 0.46, *p* = 0.51), nor a significant interaction of age and scan session (Cognitive Training: *F*_(1,16)_ = 0.72, *p* = 0.41; Walking: *F*_(1,11)_ = 0.32, *p* = 0.59) on the number of nodes with a degree >10. ***C***, Line graphs depicting the modularity of the 20 highest degree nodes for the Cognitive Training and (***D***) Walking conditions. In both conditions, there was not a main effect of age (Cognitive Training: *F*_(1,16)_ = 1.40, *p* = 0.25; Walking: *F*_(1,11)_ = 0.003, *p* = 0.96) or scan session (Cognitive Training: *F*_(1,16)_ = 0.58, *p* = 0.50; Walking: *F*_(1,11)_ < 0.001, *p* = 0.99). There was a significant interaction with age and scan session in the Cognitive Training condition (*F*_(1,16)_ = 6.21, *p* = 0.02, η^2^ = 0.28), which was not evident in the Walking condition (*F*_(1,11)_ = 0.15, *p* = 0.71). Error bars are ± 1 SEM.

Global connectivity metrics were calculated to determine whether other quantitative differences existed between age groups and experimental conditions. These metrics of global connectivity included node degree and strength, and modularity (the fraction of the edges that fall within given groups minus the expected fraction if edges were distributed at random), and global clustering coefficient (a measure of the extent to which the network exhibits a small-world structure by calculating the ratio of connected to unconnected triplets of nodes). The distributions of node degrees for young and aged rats across scan sessions is shown in Figure 4*A*,*B* for the Cognitive Training and Walking conditions, respectively. Although the distributions of node degree qualitatively suggests that there is a decrease in nodes with >10° in young rats and increase in aged rats that were cognitively trained, quantitatively, repeated-measures ANOVAs revealed that there was not a significant main effect of age (Cognitive Training: *F*_(1,16)_ = 0.70, *p* = 0.42; Walking: *F*_(1,11)_ = 0.03, *p* = 0.86), scan session (Cognitive Training: *F*_(1,16)_ = 0.40, *p* = 0.54; Walking: *F*_(1,11)_ = 0.46, *p* = 0.51), nor a significant interaction of age and scan session (Cognitive Training: *F*_(1,16)_ = 0.72, *p* = 0.41; Walking: *F*_(1,11)_ = 0.32, *p* = 0.59) on the number of nodes with a degree >10. A similar pattern was observed in the global node strength (data not shown), in which global node strength did not significantly differ between age groups in either the Cognitive Training or the Walking conditions (Cognitive Training: *F*_(1,16)_ = 0.16, *p* = 0.70; Walking: *F*_(1,11)_ = 0.01, *p* = 0.91). There was also not a main effect of scan session (Cognitive Training: *F*_(1,16)_ = 0.01, *p* = 0.93; Walking: *F*_(1,11)_ = 0.12, *p* = 0.74), nor a significant interaction of age and scan session (Cognitive Training: *F*_(1,16)_ = 0.02, *p* = 0.90; Walking: *F*_(1,11)_ = 2.55, *p* = 0.14). Furthermore, global modularity did not significantly differ between age groups in either condition (Cognitive Training: *F*_(1,16)_ = 1.83, *p* = 0.20; Walking: *F*_(1,11)_ = 0.03, *p* = 0.88), nor was there a significant interaction of age and scan session (Cognitive Training: *F*_(1,16)_ = 0.001, *p* = 0.98; Walking: *F*_(1,11)_ = 0.94, *p* = 0.35). While the main effect of scan session was not significant for the Cognitive Training condition (*F*_(1,16)_ = 1.35, *p* = 0.26), there was a significant effect for rats in the Walking condition, such that global modularity was reduced across both age groups after the second scan session (*F*_(1,11)_ = 12.35, *p* = 0.01, η^2^ = 0.53).

Similar to the measure of global modularity, the global clustering coefficient did not significantly differ with age (Cognitive Training: *F*_(1,16)_ = 0.47, *p* = 0.50; Walking: *F*_(1,11)_ = 0.01, *p* = 0.93). The interaction of age and scan session was also not significant (Cognitive Training: *F*_(1,16)_ = 0.83, *p* = 0.38; Walking: *F*_(1,11)_ = 0.00, *p* = 0.99). Again, while the main effect of scan session was not significant for the Cognitive Training rats (*F*_(1,16)_ = 0.43, *p* = 0.52), there was a significant decrease for the activity-matched controls in the global clustering coefficient after the second scan session in both young and aged rats (*F*_(1,11)_ = 8.51, *p* = 0.01, η^2^ = 0.43).

A previous study has reported that, even when global connectivity metrics did not differ between young and aged rats, nor with cognitive training, there was a significant reorganization of the resting state connectivity of the highest strength nodes (Colon-Perez et al., 2019). Given observed increase in high degree nodes across scans in the aged rats, the modularity of the 20 highest degree nodes (10 per hemisphere, given lack of evidence of unilaterality) was quantified across scan session for young and aged rats in the Cognitive Training (Fig. 4*C*) and Walking conditions (Fig. 4*D*). In both the Cognitive Training and Walking conditions, there was not a main effect of age (Cognitive Training: *F*_(1,16)_ = 1.40, *p* = 0.25; Walking: *F*_(1,11)_ = 0.003, *p* = 0.96) or scan session (Cognitive Training: *F*_(1,16)_ = 0.58, *p* = 0.50; Walking: *F*_(1,11)_ < 0.001, *p* = 0.99). While there was not a significant interaction of scan session and age in the Walking condition (*F*_(1,11)_ = 0.15, *p* = 0.71), there was a significant interaction with age and scan session in the Cognitive Training condition (*F*_(1,16)_ = 6.21, *p* = 0.02, η^2^ = 0.28). These results indicate that the modularity of these high degree nodes increased at scan 2 for aged rats, while decreasing for young rats. This same pattern was observed if modularity was calculated on the 20 highest strength nodes in which modularity of these high strength nodes increased at scan 2 for aged rats while decreasing for young rats (*F*_(1,16)_ = 9.22, *p* = 0.01, η^2^ = 0.37) that were in the Cognitive Training condition. This interaction of scan session and age was not observed in the Walking condition (*F*_(1,11)_ = 0.35, *p* = 0.57). Together, these data indicate that the subset of nodes that showed the greatest connectivity and strength in aged rats showed more modularity following cognitive training, but not walking. Because modularity reflects the degree of segregation among different nodes, this observation suggests that cognitive training in aged rats may differentiate densely connected nodes. This network reorganization is not seen in young rats under similar behavioral conditions.

### Seed-based connectivity

To assess whether age group and training condition impacted the resting state functional connectivity of brain structures that are believed to support PAL task performance, or that have been shown to have vulnerable network connectivity patterns in advanced age, seed-based correlation matrices were generated for the following regions: perirhinal cortex and dorsal hippocampus of the MTL, prelimbic and infralimbic cortices of frontal regions, and retrosplenial cortex. Connectivity between these areas was then quantified in relation to age, behavioral condition, and scan session.

**Figure 5.**
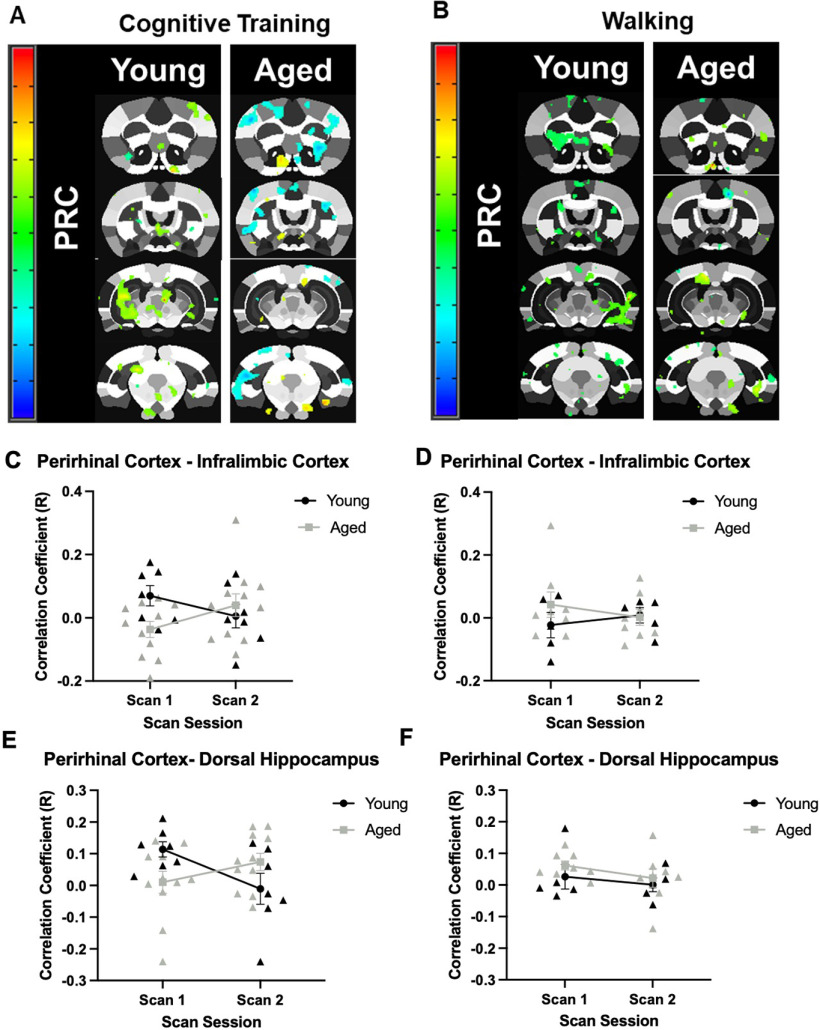
Seed-based connectivity for the perirhinal cortex. ***A***, Voxel analyses in young and aged rats across scan sessions in response to Cognitive Training (Young: *N* = 7; Aged: *N* = 11), or (***B***) Walking (Young: *N* = 5; Aged: *N* = 8). Panels ***C–F*** show the correlation coefficients of the seed-based analysis (*y*-axis) by scan session (*x*-axis). ***C***, In the Cognitive Training condition, the correlation coefficient of the perirhinal cortex and infralimbic cortex did not significantly differ by age (*F*_(1,16)_ = 1.71, *p* = 0.21) or scan session (*F*_(1,16)_ = 0.03, *p* = 0.88). The interaction between age and scan session, however, trended toward significance (*F*_(1,16)_ = 3.22 *p* = 0.09). ***D***, In the Walking condition, the correlation coefficient of the perirhinal cortex and infralimbic cortex did not significantly differ between age groups (*F*_(1,11)_ = 0.87, *p* = 0.37), scan session (*F*_(1,11)_ = 0.01, *p* = 0.91), nor was the interaction significant (*F*_(1,11)_ = 0.81, *p* = 0.39). ***E***, In the Cognitive Training condition, the correlation coefficient of the perirhinal cortex and dorsal hippocampus did not differ by age group (*F*_(1,16)_ = 0.07, *p* = 0.79) or scan session (*F*_(1,16)_ = 0.75, *p* = 0.40). The interaction between age and scan session, however, reached significance (*F*_(1,16)_ = 7.33, *p* = 0.02, η^2^ = 0.31). ***F***, In the Walking condition, the correlation coefficient of the perirhinal cortex and dorsal hippocampus was not significantly different between age groups (*F*_(1,11)_ = 1.34, *p* = 0.27), scan sessions (*F*_(1,11)_ = 1.27, *p* = 0.28), nor did the interaction effect reach significance (*F*_(1,11)_ = 0.06, *p* = 0.81). Error bars are ± 1 SEM.

Seed based connectivity maps for the perirhinal cortex are shown in Figure 5*A*,*B* for the Cognitive Training and Walking conditions. Correlation coefficients between the perirhinal cortex and the infralimbic cortex was observed to interact with age group in response to cognitive training (Fig. 5*C*). While there was not a significant main effect of age (*F*_(1,16)_ = 1.71, *p* = 0.21) or scan session (*F*_(1,16)_ = 0.03, *p* = 0.88), the interaction between age and scan session trended toward being significant (*F*_(1,16)_ = 3.22 *p* = 0.09). In the Walking condition (Fig. 5*D*), there was not a significant main effect of age (*F*_(1,11)_ = 0.87, *p* = 0.37) or scan session (*F*_(1,11)_ = 0.01, *p* = 0.91), nor a significant interaction between age and scan session (*F*_(1,11)_ = 0.81, *p* = 0.39).

Interestingly, although the correlation coefficient between the perirhinal cortex and the infralimbic cortex differed between young rats in the Cognitive Training and Walking conditions at scan 1, both demonstrated near 0.0 correlation coefficients at scan 2. In contrast, while aged rats also demonstrated different correlation coefficients across conditions at scan 1, rats in the Cognitive Training condition demonstrated an increase in correlation between the perirhinal cortex and the infralimbic cortex at scan 2, while the correlation coefficient hovered around 0.0 for rats in the Walking condition.

A similar pattern was observed for the correlation coefficient between perirhinal cortex and dorsal hippocampus in young and aged rats across scan sessions. There was not a significant main effect of age (*F*_(1,16)_ = 0.07, *p* = 0.79) or scan session (*F*_(1,16)_ = 0.75, *p* = 0.40), but there was a significant interaction between age and scan session (*F*_(1,16)_ = 7.33, *p* = 0.02, η^2^ = 0.31) in the Cognitive Training condition (Fig. 5*E*). For the Walking condition, there was also not a significant main effect of age (*F*_(1,11)_ = 1.34, *p* = 0.27) or scan session (*F*_(1,11)_ = 1.27, *p* = 0.28; Fig. 5*F*). Unlike the rats that underwent cognitive training, there was not a significant interaction between age and scan session for the Walking condition (*F*_(1,11)_ = 0.06, *p* = 0.81).

Of note, although the correlation coefficient between the perirhinal cortex and the dorsal hippocampus differed between young rats in the Cognitive Training and Walking conditions at scan 1, both demonstrated near 0.0 correlation coefficients at scan 2. This was not the case for aged rats across conditions, who differed in their correlation coefficient at scan 2.

**Figure 6.**
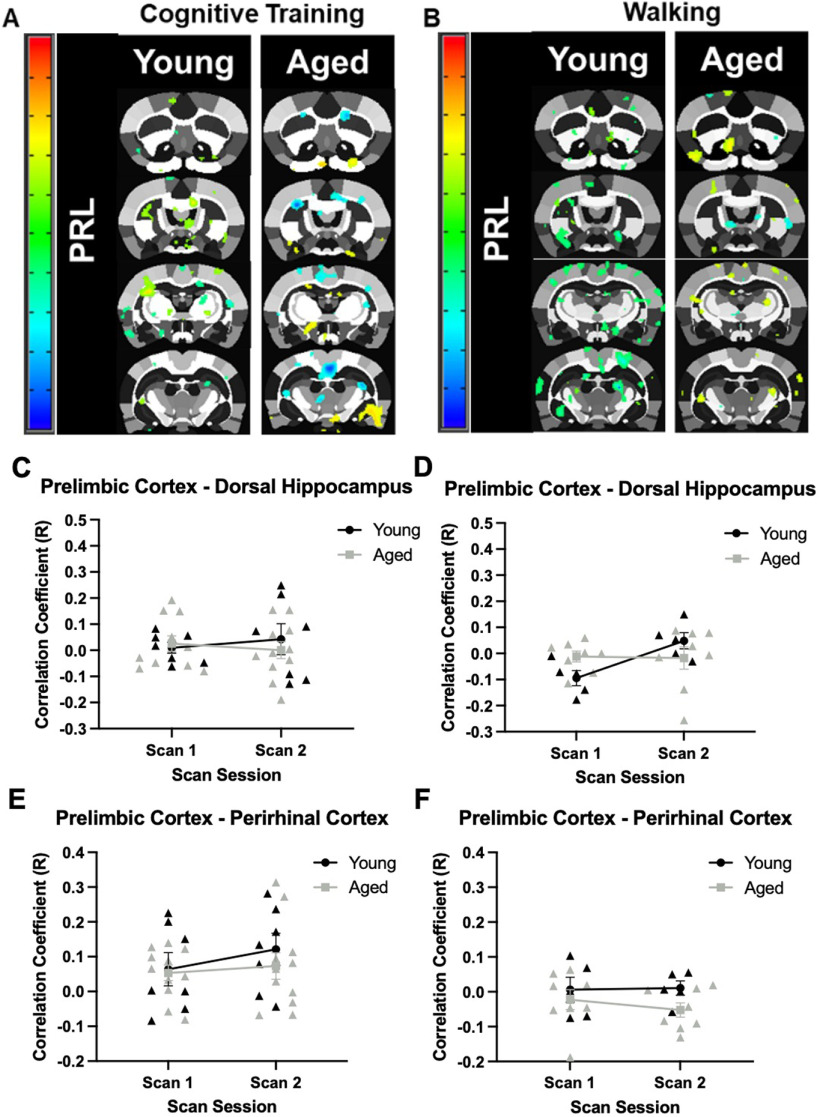
Seed-based connectivity for the prelimbic cortex. ***A***, Whole-brain voxel analyses in young and aged rats across scan sessions for the Cognitive Training (Young: *N* = 7; Aged: *N* = 11), and (***B***) Walking conditions (Young: *N* = 5; Aged: *N* = 8). Panels ***C–F*** show the correlation coefficients (*y*-axis) by scan session (*x*-axis). ***C***, In the Cognitive Training condition, the correlation coefficient of the prelimbic cortex and dorsal hippocampus did not significantly differ by age (*F*_(1,16)_ = 0.15, *p* = 0.70), scan session (*F*_(1,16)_ = 0.01, *p* = 0.93), nor was the interaction significant (*F*_(1,16)_ = 0.51, *p* = 0.49). ***D***, In the Walking condition, the correlation coefficient of the prelimbic cortex and dorsal hippocampus also did not differ between age groups (*F*_(1,11)_ = 0.10, *p* = 0.76), scan sessions (*F*_(1,11)_ = 2.79, *p* = 0.12), nor was there and a significant interaction (*F*_(1,11)_ = 3.32, *p* = 0.10). ***E***, The correlation coefficient of the prelimbic cortex and perirhinal cortex in young and aged rats in the Cognitive Training condition did not significantly differ between age groups (*F*_(1,16)_ = 0.54, *p* = 0.47), scan sessions (*F*_(1,16)_ = 1.13, *p* = 0.30), nor was there a significant interaction (*F*_(1,16)_ = 0.27, *p* = 0.61). ***F***, In the Walking condition, the correlation coefficient of the prelimbic cortex and perirhinal cortex in young and aged rats did not differ between age groups (*F*_(1,11)_ = 4.86, *p* = 0.05, η^2^ = 0.31), scan sessions (*F*_(1,11)_ = 0.14, *p* = 0.71), nor was the interaction significant (*F*_(1,11)_ = 0.27, *p* = 0.61). Error bars are ±1 SEM.

Overall, the prelimbic cortex showed low correlations with other regions, suggesting limited resting state functional connectivity with the regions of interest (Fig. 6*A*,*B*) for both age groups and training conditions. Moreover, the patterns of prelimbic functional connectivity did not vary by scan session. Specifically, the correlation coefficient for the prelimbic cortex and dorsal hippocampus in young and aged rats did not significantly vary between age groups (*F*_(1,16)_ = 0.15, *p* = 0.70; Fig. 6*C*), scan sessions (*F*_(1,16)_ = 0.01, *p* = 0.93), nor was there a significant interaction of age and scan session (*F*_(1,16)_ = 0.51, *p* = 0.49) in the Cognitive Training condition. For Walking condition (Fig. 6*D*), there was also not a significant effect of age (*F*_(1,11)_ = 0.10, *p* = 0.76), scan session (*F*_(1,11)_ = 2.79, *p* = 0.12), nor an interaction of age and scan session (*F*_(1,11)_ = 3.32, *p* = 0.10). In the Cognitive Training condition, the correlation coefficient between the prelimbic and perirhinal cortices (Fig. 6*E*) also did not significantly vary between age groups (*F*_(1,16)_ = 0.54, *p* = 0.47), scan sessions (*F*_(1,16)_ = 1.13, *p* = 0.30), nor was the interaction of age and scan session significantly different (*F*_(1,16)_ = 0.27, *p* = 0.61). For the Walking group (Fig. 6*F*), while there was a significant main effect of age group, with aged rats having lower correlation values overall (*F*_(1,11)_ = 4.86, *p* = 0.05, η^2^ = 0.31), there was not a significant effect of scan session (*F*_(1,11)_ = 0.14, *p* = 0.71), nor a significant interaction of scan session and age (*F*_(1,11)_ = 0.27, *p* = 0.61).

**Figure 7.**
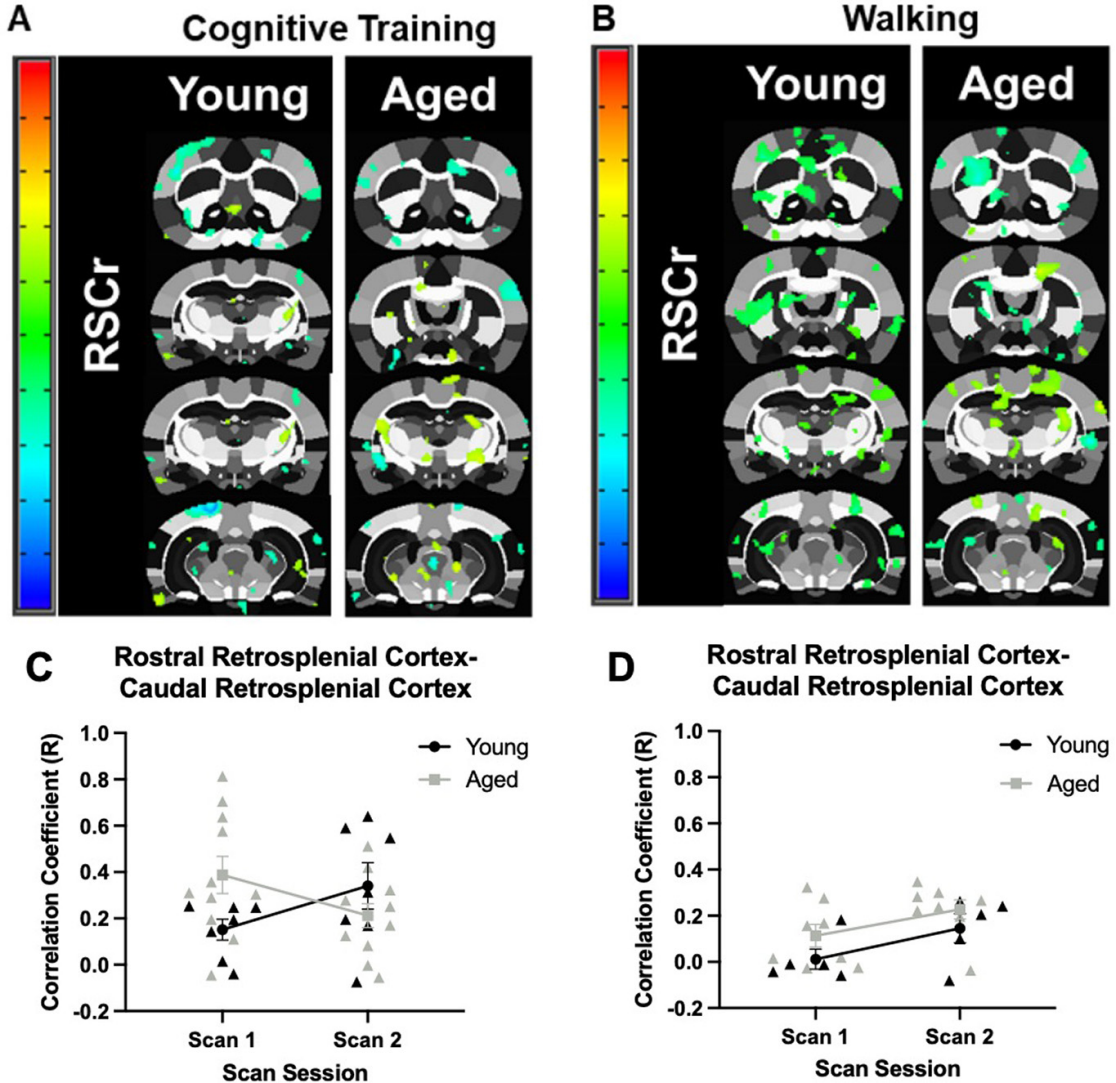
Age-related differences in seed-based connectivity for the rostral retrosplenial cortex. ***A***, Rostral retrosplenial cortex to whole-brain voxel analyses in young and aged rats across scan sessions in response to Cognitive Training (Young: *N* = 7; Aged: *N* = 11), or (***B***) Walking (Young: *N* = 5; Aged: *N* = 8). Panels ***C***, ***D*** show the correlation coefficients (*y*-axis) by scan session (*x*-axis). ***C***, In the Cognitive Training condition, the correlation coefficient of the rostral and caudal retrosplenial cortex did not significantly differ between age groups (*F*_(1,16)_ = 0.59, *p* = 0.46), or scan sessions (*F*_(1,16)_ = 0.01, *p* = 0.93). There was, however, a significant interaction of age and scan session (*F*_(1,16)_ = 5.43, *p* = 0.03, η^2^ = 0.25). ***D***, In the Walking condition, the correlation coefficient of the rostral and caudal retrosplenial cortex was significantly different between scan sessions (*F*_(1,11)_ = 9.52, *p* = 0.01, η^2^ = 0.46), but there was not a significant effect of age group (*F*_(1,11)_ = 2.40, *p* = 0.15). Unlike the rats that underwent Cognitive Training, there was not a significant interaction between age and scan session (*F*_(1,11)_ = 0.07, *p* = 0.80). Error bars are ±1 SEM.

Connectivity maps for the rostral retrosplenial cortex are shown in Figure 7*A*,*B*. This area showed the greatest connectivity with the caudal retrosplenial cortex. When the correlation coefficient between the rostral and caudal retrosplenial cortex in young and aged rats was evaluated across scan sessions in response to cognitive training (Fig. 7*C*), there was not a significant main effect of age (*F*_(1,16)_ = 0.59, *p* = 0.46) or scan session (*F*_(1,16)_ = 0.01, *p* = 0.93). There was, however, a significant interaction of age and scan session (*F*_(1,16)_ = 5.43, *p* = 0.03, η^2^ = 0.25) in response to cognitive training. For the Walking condition (Fig. 7*D*), there was a significant main effect of scan session (*F*_(1,11)_ = 9.52, *p* = 0.01, η^2^ = 0.46), but not a significant effect of age (*F*_(1,11)_ = 2.40, *p* = 0.15). Unlike the rats that underwent cognitive training, there was not a significant interaction between age and scan session (*F*_(1,11)_ = 0.07, *p* = 0.80). This suggests that, unlike the Cognitive Training condition, functional connectivity between the caudal and rostral retrosplenial cortex increased in response to walking for reward in both age groups. While the correlation coefficient between the rostral and caudal retrosplenial cortex did not differ greatly at scan 1 between young rats in either condition, the correlation coefficient did differ for aged rats across conditions and became more similar at scan 2.

### Clustering coefficients

**Figure 8.**
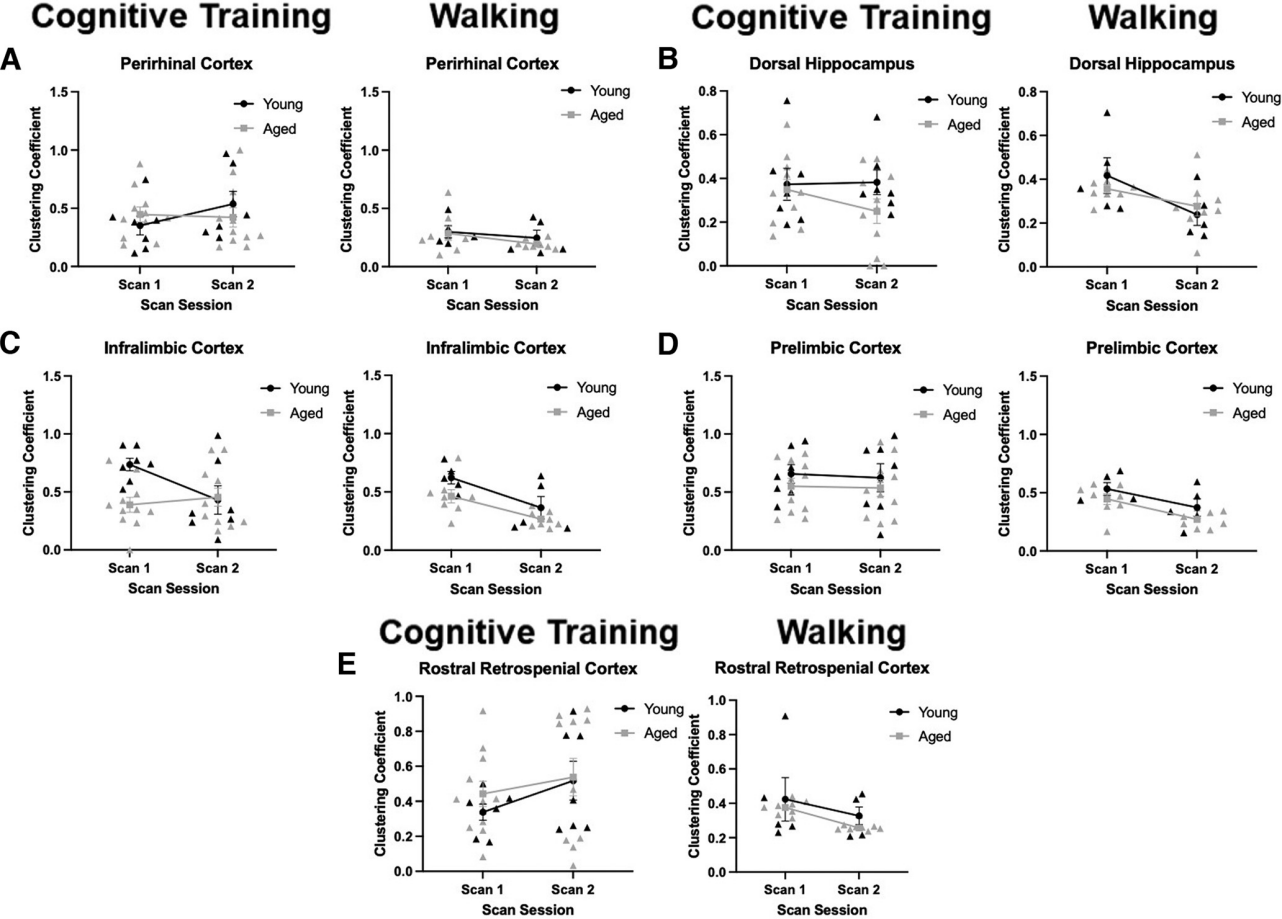
Clustering coefficients. Panels ***A–E*** show the clustering coefficient (*y*-axis) by scan session (*x*-axis) for the Cognitive Training (left panels; Young: *N* = 7; Aged: *N* = 11) and Walking (right panels; Young: *N* = 5; Aged: *N* = 8) conditions. ***A***, In the perirhinal cortex, there was not a significant effect of age (Cognitive Training: *F*_(1,16)_ = 0.02, *p* = 0.90; Walking: *F*_(1,11)_ = 0.40, *p* = 0.54), scan session (Cognitive Training: *F*_(1,16)_ = 0.72, *p* = 0.41; Walking: *F*_(1,11)_ = 2.06, *p* = 0.18), nor a significant interaction (Cognitive Training: *F*_(1,16)_ = 1.24, *p* = 0.28; Walking: *F*_(1,11)_ = 0.15, *p* = 0.71). ***B***, In the dorsal hippocampus, there was not a significant main effect of age (Cognitive Training: *F*_(1,16)_ = 2.43, *p* = 0.14; Walking: *F*_(1,11)_ = 0.03, *p* = 0.86) nor a significant interaction (Cognitive Training: *F*_(1,16)_ = 0.68, *p* = 0.42; Walking: *F*_(1,11)_ = 2.24, *p* = 0.16). There was not a significant effect of scan session for Cognitive Training (*F*_(1,16)_ = 0.47, *p* = 0.50), but there was a significant effect of scan session in the Walking condition (*F*_(1,11)_ = 14.94, *p* = 0.003, η^2^ = 0.58). ***C***, In the infralimbic cortex, for the Cognitive Training condition, there was a significant effect of age (*F*_(1,16)_ = 4.47, *p* = 0.05, η^2^ = 0.22) and an interaction of scan session and age (*F*_(1,16)_ = 4.43, *p* = 0.05, η^2^ = 0.22). There was not a significant effect of scan session (*F*_(1,16)_ = 1.84, *p* = 0.19). In the Walking condition, there was a significant effect of age (*F*_(1,11)_ = 5.31, *p* = 0.04, η^2^ = 0.33) and an effect of scan session (*F*_(1,11)_ = 14.22, *p* = 0.003, η^2^ = 0.56). Unlike the Cognitive Training rats, there was not a significant interaction of age and scan session (*F*_(1,11)_ = 0.26, *p* = 0.62). ***D***, In the prelimbic cortex, there was not a significant main effect of age (Cognitive Training: *F*_(1,16)_ = 1.73, *p* = 0.21; Walking: *F*_(1,11)_ = 3.54, *p* = 0.09) or an interaction of scan session and age (Cognitive Training: *F*_(1,16)_ = 0.01, *p* = 0.92; Walking: *F*_(1,11)_ = 0.02, *p* = 0.89). While there was not an effect of scan session for the Cognitive Training condition (*F*_(1,16)_ = 0.07, *p* = 0.80), there was a significant decrease in the Walking condition (*F*_(1,11)_ = 11.66, *p* = 0.01, η^2^ = 0.51). ***E***, In the rostral retrosplenial cortex, there was not a significant main effect of age (Cognitive Training: *F*_(1,16)_ = 0.50, *p* = 0.49; Walking: *F*_(1,11)_ = 2.39, *p* = 0.15), scan session (Cognitive Training: *F*_(1,16)_ = 1.91, *p* = 0.19; Walking: *F*_(1,11)_ = 2.75, *p* = 0.13), nor an interaction of scan session and age (Cognitive Training: *F*_(1,16)_ = 0.18, *p* = 0.67; Walking: *F*_(1,11)_ = 0.03, *p* = 0.87). Error bars are ±1 SEM.

The local clustering coefficient of regions of interest (perirhinal cortex, dorsal hippocampus, infralimbic cortex, prelimbic cortex, and rostral retrosplenial cortex) was quantified by scan session and age (Fig. 8) for the Cognitive Training (left panels) and Walking (right panels) conditions. For each of these regions of interest, the local clustering coefficient for that region would indicate the probability that any two functionally connected nodes with that structure would also be connected to each other. Thus, higher clustering coefficients indicate the extent to which a region participates in a small world network or rich club architecture. For the perirhinal cortex, in both Cognitive Training and Walking conditions, there was not a significant main effect of age (Cognitive Training: *F*_(1,16)_ = 0.02, *p* = 0.90; Walking: *F*_(1,11)_ = 0.40, *p* = 0.54), scan session (Cognitive Training: *F*_(1,16)_ = 0.72, *p* = 0.41; Walking: *F*_(1,11)_ = 2.06, *p* = 0.18), nor a significant interaction of scan session and age (Cognitive Training: *F*_(1,16)_ = 1.24, *p* = 0.28; Walking: *F*_(1,11)_ = 0.15, *p* = 0.71; Fig. 8*A*).

For the dorsal hippocampus, in both the Cognitive Training and Walking conditions, there was not a significant main effect of age (Cognitive Training: *F*_(1,16)_ = 2.43, *p* = 0.14; Walking: *F*_(1,11)_ = 0.03, *p* = 0.86) nor a significant interaction of scan session and age (Cognitive Training: *F*_(1,16)_ = 0.68, *p* = 0.42; Walking: *F*_(1,11)_ = 2.24, *p* = 0.16; Fig. 8*B*). While there was not a significant main effect of scan session for rats that underwent cognitive training (*F*_(1,16)_ = 0.47, *p* = 0.50) there was a significant effect of scan session in the Walking condition (*F*_(1,11)_ = 14.94, *p* = 0.003, η^2^ = 0.58), with both age groups showing reduced clustering in the second scan.

In the infralimbic cortex, the clustering coefficient across scan sessions showed different patterns between the Cognitive Training and Walking conditions that interacted with age group. For the Cognitive Training condition (Fig. 8*C*, left panel), the aged rats had a significantly lower clustering coefficient (*F*_(1,16)_ = 4.47, *p* = 0.05, η^2^ = 0.22), but there was not a significant main effect of scan session (*F*_(1,16)_ = 1.84, *p* = 0.19). There was, however, a significant interaction of scan session and age (*F*_(1,16)_ = 4.43, *p* = 0.05, η^2^ = 0.22). This was because of the clustering coefficient decreasing between scan sessions in the young animals, but not the aged rats, that underwent cognitive training. In the Walking condition (Fig. 8*C*, right panel), there was a significant main effect of age (*F*_(1,11)_ = 5.31, *p* = 0.04, η^2^ = 0.33) and scan session (*F*_(1,11)_ = 14.22, *p* = 0.003, η^2^ = 0.56). Unlike the cognitive training rats, there was not a significant interaction of age and scan session for activity-matched controls (*F*_(1,11)_ = 0.26, *p* = 0.62), and the infralimbic cortex in both young and aged rats had lower local clustering coefficient values. Together, these data suggest that the aged infralimbic cortex has reduced rich club participation compared with young animals, but this can be modulated by cognitive training. Because the young rats showed reductions in the local clustering coefficient values of the infralimbic cortex in both conditions, but the aged rats only expressed this pattern following walking, these data may reflect a decline in rich club participation of the infralimbic cortex as a task becomes more familiar or less cognitively challenging. In aged rats with PAL impairments, this metric of network organization does not change, as the task may require a significant cognitive load even after criterion has been achieved.

The clustering coefficient values for the prelimbic cortex did not significantly differ by age group for either the Cognitive Training or Walking conditions (Cognitive Training: *F*_(1,16)_ = 1.73, *p* = 0.21; Walking: *F*_(1,11)_ = 3.54, *p* = 0.09), nor was there an interaction of scan session and age (Cognitive Training: *F*_(1,16)_ = 0.01, *p* = 0.92; Walking: *F*_(1,11)_ = 0.02, *p* = 0.89; Fig. 8*D*). While there was not a main effect of scan session for Cognitive Training rats (*F*_(1,16)_ = 0.07, *p* = 0.80), there was a significant effect for the Walking condition (*F*_(1,11)_ = 11.66, *p* = 0.01, η^2^ = 0.51), with clustering declining between scan sessions in both age groups, similar to what was observed for the infralimbic cortex.

In the rostral retrosplenial cortex in both the Cognitive Training and Walking conditions, there was not a significant main effect of age (Cognitive Training: *F*_(1,16)_ = 0.50, *p* = 0.49; Walking: *F*_(1,11)_ = 2.39, *p* = 0.15), scan session (Cognitive Training: *F*_(1,16)_ = 1.91, *p* = 0.19; Walking: *F*_(1,11)_ = 2.75, *p* = 0.13), nor an interaction of scan session and age (Cognitive Training: *F*_(1,16)_ = 0.18, *p* = 0.67; Walking: *F*_(1,11)_ = 0.03, *p* = 0.87; Fig. 8*E*).

### Associations of resting state functional connectivity and behavioral variables

Given changes identified in global and seed-based functional connectivity following cognitive training, a stepwise linear regression was performed to determine whether PAL task performance at day 15 was predicted by a change in any of the functional connectivity variables for which there were significant interactions between age groups and scan sessions. Performance at day 15 was selected, as this is the day of testing in which the performance difference between young and aged rats was the largest. The resting state functional connectivity variables in the model included: change in the number of edges with *z *> 0.3, modularity of the 20 highest degree nodes, modularity of the 20 highest strength nodes, connectivity between the perirhinal cortex and infralimbic cortex, connectivity between the perirhinal cortex and dorsal hippocampus, connectivity between the rostral and caudal restrosplenial cortex, infralimbic cortex clustering coefficient, and rostral retrosplenial cortex clustering coefficient. All variables were transformed into *z* scores calculated from the animals’ respective age group distribution.

**Figure 9.**
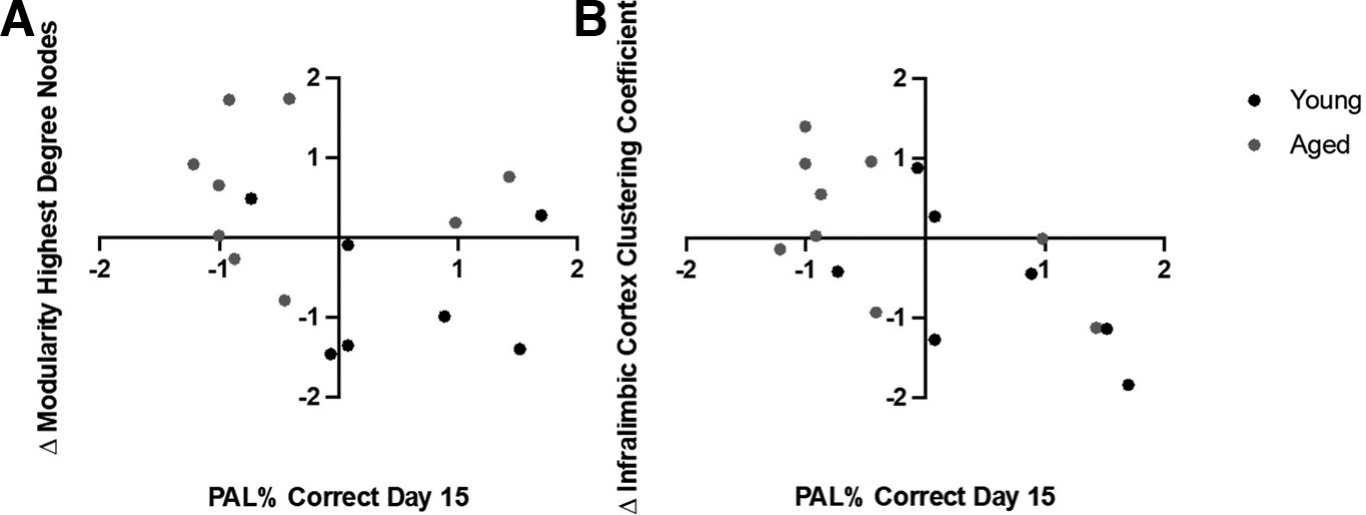
Relationship of resting state connectivity to PAL performance. ***A***, Scatter plot representing the *z* score normalized relationship of PAL percent accuracy at day 15 and modularity of the 20 highest degree nodes across scan sessions in all rats (Young: *N* = 7; Aged: *N* = 9). Stepwise linear regression revealed that change in modularity of the 20 highest degree nodes significantly predicted PAL performance at day 15 ( B = −0.49, *t* = −3.02, *p* = 0.01). ***B***, Scatter plot representing the relationship of PAL percent accuracy at day 15 and change in infralimbic cortex clustering coefficient across scan sessions in all rats. Stepwise linear regression revealed that change in infralimbic cortex clustering coefficient significantly predicted PAL performance at day 15 ( B = −0.81, *t* = −4.71, *p* < 0.001).

The final stepwise linear regression model was significant (*F*_(1,13)_ = 13.3, *p* = 0.001), explaining 62.1% of the variance in PAL task percent accuracy at day 15 (adjusted *R*^2^ = 0.62). Functional connectivity variables that significantly predicted behavioral task performance included the change in modularity of the highest degree nodes and change in infralimbic cortex clustering coefficient (modularity of highest degree nodes: β = −0.49, *t* = −3.02, *p* = 0.01; infralimbic cortex clustering coefficient: β = −0.81, *t* = −4.71, *p* < 0.001; Fig. 9*A*,*B*). Specifically, increased modularity of highest degree nodes and increased infralimbic cortex rich club participation at scan 2 was inversely related to task performance at day 15. In other words, the rats that showed the largest change in connectivity metrics were those that performed the worst after two weeks of training. There are two mutually exclusive explanations that may account for this observation. First, it is possible that greater levels of global network reorganization were necessary for these animals to reach criterion performance, but the altered functional connectome ultimately facilitated appropriate behavior on the PAL task. Alternatively, the change in network connectivity could reflect connectome instability that impedes PAL task acquisition. Future experiments will be necessary to disentangle these competing hypotheses.

## Discussion

### Summary of results

The present study documented the extent to which cognitive training with a translational, touchscreen-based paired-associates learning (PAL) task resulted in distinct changes in global and seed-based resting state functional connectivity in aged compared with young rats. All rats were shaped to nose poke a touchscreen monitor to retrieve a food reward and then completed an initial resting state fMRI scan under light anesthesia. Rats were then trained on the PAL task, and a second resting state scan was completed once criterion was achieved. It was observed that, following cognitive training on the PAL task, there were several alterations in resting state functional connectivity that were only observed in the aged rats. These changes included: (1) an increase in the total number of significant degrees in the network, (2) enhanced modularity of high degree and high strength nodes, (3) increased connectivity between the perirhinal and infralimbic cortices, (4) increased connectivity between the perirhinal cortex and dorsal hippocampus, (5) increased connectivity between the rostral and caudal retrosplenial cortex, and (6) increased local clustering coefficient for the infralimbic cortex. These resting state functional connectivity alterations were selective to aged rats that underwent cognitive training, as resting state network changes across scan sessions were not observed in young rats. Moreover, for both young and aged rats that traversed a track daily for rewards (the Walking condition) and were not cognitively trained between resting state scans, significant changes in resting state functional connectivity between scans were not observed. Broadly, these observations suggest that functional connectome organization, as inferred from inter-regional correlations in the BOLD signal, in old animals is not a fixed architecture, but rather a dynamic network that can be updated by training that involves a cognitive load.

### Comparison to similar studies

Consistent with the observations reported by Colon-Perez et al. (2019), the present study identified increased network connectivity in aged rats following cognitive training. The subnetwork in which Colon-Perez et al. (2019) identified an increase in node strength and degree in task-relevant regions increased as a function of cognitive training. In the present study, several indicators of increased network connectivity were identified. Specifically, edge weights were represented as *z* scores, normalized such that all edge weights had values between 0 and 1 to minimize the impact of between-subject variability (Colon-Perez et al., 2018). Greater edge weights (*z* ≥ 0.3) represent more highly connected nodes; therefore, an increase in the number of edges *z* ≥ 0.3 at scan 2 in aged rats suggests an increase in highly connected nodes. This relationship was not identified in rats that underwent a control walking task, and young rats that completed the cognitive training task displayed a decrease in the number of edges *z* ≥ 0.3 at scan 2. Additionally, modularity is an indicator of the degree of segregation across nodes. The present study identified increased subnetwork modularity in aged rats following cognitive training and not young rats or Walking controls. Because modularity reflects the degree of segregation among different nodes, this observation suggests that cognitive training in aged rats may differentiate densely connected nodes. This network reorganization is not seen in young rats under similar behavioral conditions.

An important consideration is whether alterations in functional connectivity were related to behavioral performance. While a causative relationship between resting state connectivity and cognition cannot be established with the current data, increases in modularity and clustering coefficient in the infralimbic cortex were both correlated with PAL performance. Specifically, rats with worse performance after two weeks of training were the rats that showed the greatest enhancement in modularity and local clustering of the infralimbic cortex after performance criterion had been reached. This observation points to two distinct possibilities regarding the relationship between resting state functional connectivity organization and PAL performance. One hypothesis is that changes in resting state connectivity that occur over the course of cognitive training reflect network instability that delay an aged animal’s ability to achieve optimal performance. An alternative and mutually exclusive hypothesis is that changes in resting state connectivity reflect a compensatory network reorganization that is greater among aged rats with the largest impairments. These more severely impaired aged animals may need greater network reorganization to reach criterion levels of behavior. If the former is true, then manipulations that stabilize the resting state connectome will improve behavior. If the latter is true, more dynamic resting state connectivity would facilitate cognitive resilience during aging. Additional experiments will be necessary to disentangle these mutually exclusive possibilities.

Previous studies have also linked age-related differences in functional connectivity to cognitive performance in rats. The first study to do this characterized aged rats as impaired or unimpaired on the Morris watermaze and then compared resting state functional connectivity between young, aged-impaired, and aged-unimpaired rats (Ash et al., 2016). Using a seed-based functional connectivity analysis centered on retrosplenial/posterior parietal cortex, all rats had patterns of connectivity that were consistent with the default mode network that has been reported in young rats (Lu et al., 2012), monkeys (Mantini et al., 2011), and humans (Greicius et al., 2003; Fox et al., 2009; Mormino et al., 2011). In the aged-impaired rats only, however, functional connectivity between the retrosplenial cortex and sensorimotor cortex, posterior parietal/secondary visual cortex, and dorsal auditory/temporal association cortex was reduced relative to aged unimpaired and young animals. Resting state functional connectivity was similar between the young and aged-unimpaired rats (Ash et al., 2016). A more recent seed-based analysis that was focused on the CA1 and CA3 subregions of the hippocampus reported that resting state functional connectivity was lower in aged-impaired rats compared with young animals between these hippocampal regions and the orbitofrontal cortices, prelimbic and infralimbic cortices, other medial temporal lobe (MTL) structures, and left temporal association cortex (Liang et al., 2020). In both the Ash et al. (2016) and Liang et al. (2020) experiments, resting state fMRI scans were obtained approximately onoe month after Morris watermaze training. Thus, it is unlikely that the training influenced resting state functional connectivity, and these data may be comparable to the Scan 1 connectivity in the current experiment. In line with this idea, there are several observations that are consistent across these three studies, including reduced connectivity of the retrosplenial cortex, and reduced connectivity between MTL structures and infralimbic cortex. In the current study, however, the latter was observed between the infralimbic and perirhinal cortices. Unfortunately, the current study did not have the spatial resolution to examine different hippocampal subregions separately as previous work has done (Liang et al., 2020).

A unique aspect of the current study was that resting state functional connectivity was examined longitudinally in rats that were either cognitively trained on the PAL task or walked around a track for food reward during the intervening weeks between scan sessions. Longitudinal interrogation of resting state connectivity has been used with human study participants to examine changes in network structure in relation to cognitive aging or neurodegenerative disease over the course of years (H. Li et al., 2018; W. Li et al., 2020; Lin et al., 2020; de Dieu Uwisengeyimana et al., 2022). In some of these studies, enhanced functional connectivity over time was associated with cognitive resilience (Lin et al., 2020). The underlying assumptions of these investigations are that changes in functional connectivity over time are associated with the progression of cognitive aging or an underlying neurodegenerative process. The current data and other published results may challenge this assumption. It has been documented that within individual variability in functional connectivity can be large (Noble et al., 2017). While increasing scan duration can reduce some of this intraindividual variability, it is also well documented that behavioral variabilities can alter resting state functional connectivity. Motor sequence learning is associated with decreased functional connectivity across motor networks over days in normal adults (Jäger et al., 2022). Two months of meditation training leads to changes in resting state functional connectivity between the default mode and dorsal attention networks (Zhang et al., 2021). In older adults, glucose administration with a sugary drink increases functional connectivity between the posterior hippocampus (homologous to rodent dorsal hippocampus) and the medial prefrontal cortex in older adults but decreases connectivity between these structures in younger adults (Peters et al., 2020). Taken together, these data may highlight that resting state functional networks can be modified. Whether these modifications can then be reflected as improvements in cognitive output requires further investigation.

### Functional connectivity differences at scan 1 by condition

It was observed that differences in several resting state functional connectivity metrics were different between the Cognitive Training and Walking conditions at scan 1, particularly in the young rats. This could be because of the difference in activities completed by rats in each condition before scan 1. Although rats in both Cognitive Training and Walking conditions were food restricted, requiring handling and regular weighing, in the Cognitive Training condition the first scan was acquired after completion of all PAL shaping procedures. Shaping (Magazine, Any Touch, Must Touch, Must Initiate, and Punish Incorrect) required extensive procedural training, involving introduction of the rat to the touchscreen chamber, training association of a liquid reward and interaction with the touchscreen, training specific association of a liquid reward and interaction with stimuli shown on the touchscreen, trial initiation, and punishment in response to an inappropriate interaction with the touchscreen. In contrast, rats in the Walking condition traversed a circular track for 30–45 min daily, 32 laps/d, for 11 d before the first scan. Although not as cognitively demanding as the PAL test phase, the PAL shaping and behavioral procedures are likely more cognitively demanding than the Walking condition, which could have provoked differences in resting state connectivity.

Connectivity differences between food restricted young animals with no training compared with food restricted young animals with training suggests that young brains also respond to cognitive training with the PAL task, albeit differently and to different parts of the cognitive training procedure than aged rats. These changes could become more apparent if we examined a different time point; specifically, if we completed an additional scan before shaping procedures took place and compared with scans 1 and 2. Future research is needed to further explore the modifiability of network architecture in the young brain, including what cognitive factors and experiences provoke responses in the young brain. However, the Walking condition remains an appropriate comparison condition to understand how young and aged brains differentially respond to cognitive training, compared with how rats respond to a consistent walking activity that did not involve a significant cognitive load. It is clear from our study that the aged brain responds to complex cognitive training distinctively when compared with minimal change observed after daily walking or when compared with young animals in both conditions.

### Study limitations

As was previously mentioned, because of the lack of availability of female rats of this strain at the time of these experiments, sex as a biological variable could not be considered. The use of only male rats is a significant study limitation given existing evidence of differences in cognitive aging and physical abilities by sex in rodents and humans (Miller and Halpern, 2014; Hernandez et al., 2020a). Additionally, there is some evidence of sex-specific effects of cognitive and physical training in older adults (Talboom et al., 2014; Casaletto et al., 2020). Future analyses incorporating female rats are critical and could reveal different relationships of PAL task training and functional connectivity.

An additional limitation is the use of sedation during scanning. Although the use of MRI and tablet-based cognitive testing enhances the translatability of the study paradigm to human participants, humans are less typically scanned in a sedative state. It is true that inhalation anesthetic agents, such as isoflurane, can cause vasodilation and suppress neural activity. However, isoflurane is still preferred over intravenous injectable agents because of fast recovery, lower mortality, and ease of control of sedative levels in the blood. BOLD activation patterns have been successfully measures with low levels of anesthetic in animals (Masamoto et al., 2007; Kannurpatti et al., 2008; T. Kim et al., 2010; Williams et al., 2010) and humans (Riehl et al., 2018). Although functional neuroimaging in rodents can be completed in awake, head-fixed animals (Febo, 2011; Stenroos et al., 2018), acclimation to head restraint can result in chronic stress. This is important to consider given age-related differences hypothalamic-pituitary-adrenal (HPA) axis function and the contribution of HPA axis dysfunction to age-related diseases (Gupta and Morley, 2014).

Finally, while the present study does elucidate large-scale changes in functional connectome organization, it remains unclear how these network changes map onto age-associated neurobiological changes at the cellular level. In human studies, altered network connectivity in older adults is presumed to reflect neural inefficiency or dedifferentiation (Salami et al., 2014; Sala-Llonch et al., 2015; Grady et al., 2016; Nyberg, 2017). In rodent studies, it is possible to probe such relationships using noninvasive, single-cell imaging of neuron activity (Colon-Perez et al., 2019; Xu et al., 2022). For example, Colon-Perez et al. (2019) explored the relationship between response bias, increased subnetwork connectivity, and *Arc* expression in the dorsal striatum in aged rodents. Future studies exploring the relationship between tablet-based cognitive training and altered functional connectivity could examine neuron activity at the cellular level to further explore the cellular mechanisms underlying response to cognitive training in the aged brain.

## Synthesis

Reviewing Editor: Christie Fowler, University of California, Irvine Decisions are customarily a result of the Reviewing Editor and the peer reviewers coming together and discussing their recommendations until a consensus is reached. When revisions are invited, a fact-based synthesis statement explaining their decision and outlining what is needed to prepare a revision will be listed below. The following reviewer(s) agreed to reveal their identity: Yasmine Sherafat.

Experts in the field reviewed and discussed the manuscript. Both evaluated the work very positively. It was noted that the results indicate several distinct changes to whole brain networks and seed based analyses are conducted to identify specific network changes to retrosplenial and related hippocampal circuits. The primary novelty is that the results identify specific network changes in aged animals tested in the paired-associate task that have not been previously reported. This work will be of interest to a wide community of researchers investigating cognitive and neural changes in normal aging. It was also noted that the study advances the field such that the techniques for both examining brain activity (fMRI) as well as the adapted rodent task (PAL) is heavily translational as both of these are used in the clinical population.

However, several concerns were noted that should be addressed in a resubmission. In addition to the below comments, a limitations paragraph in the discussion should also include consideration of the implications with only studying male subjects.

Reviewer 1:

The authors present a very interesting study which conducted a longitudinal assessment of brain networks before and after paired associate learning (PAL). The senior author has published several important studies demonstrating that PAL is a sensitive biomarker of cognitive impairment in preclinical rat models of normal aging and the behavior is dependent on intact hippocampal-cortical circuitry. The authors investigate the dynamic changes to this circuitry using small animal neuroimaging before and after training. A similar longitudinal assessment is provided for animals that have been trained to locomote a circular track for reward (without PAL). The results indicate several distinct changes to whole brain networks and seed based analyses are conducted to identify specific network changes to retrosplenial and related hippocampal circuits. The results identify specific network changes in aged animals that will be of interest to a wide community of researchers investigating cognitive and neural changes in normal aging. The manuscript is very well written and the results are clearly presented. The analyses are appropriate. Overall, the manuscript is excellent. I only have a few minor comments for consideration.

1.) Effect sizes should be added for each significant effect in the cognitive training section.

2.) The control condition for the cognitive studies is a locomotor condition where the animals walk in a small circular environment to match the PAL condition. Locomotion and PAL was matched in time but did the authors directly compare the amount of locomotion by each animal (cumulative distance travelled)? The authors could add some additional analyses to describe the amount of activity during these sessions (e.g., number of laps or total distance travelled).

3.) Figure 3. It is interesting that there is a completely different pattern of whole brain connectivity results for the animals testing in PAL vs. the control walking condition. The difference is especially striking at scan 1 prior to PAL training (when shaping has only been conducted), and the pattern of results is more similar after PAL training. It would interesting to compare measures across the two conditions throughout the results section and if appropriate emphasize some of these differences in the discussion section.

4.) Figure 4C,D - A rationale should be presented as to why the top 20 highest degree nodes were selected for these analyses.

Reviewer 2:

1. The limitations for the implications of these findings should be elaborated on in the discussion, especially since the animals are in a sedative state during the fMRI imaging process. There was only one sentence referencing previous studies of sedation during fMRI BOLD imaging in both human and rodent studies, however, this needs to be elaborated on. Did these studies look at resting state following a task? Particularly a learning and memory task? How are the results in this study confounded by the fact that animals had to go under sedation during the fMRI task? What are the implications of these confounds when we want to think of this work for translational purposes? Alternatively, these experiments could be redone without the use of anesthesia (Stenroos et al 2018). If that’s not possible, then a more elaborate discussion on the limitations and significant alterations on the effects of anesthesia on resting state functional connectivity should be discussed.

2. For the figures, please show each subject represented on the bar graphs and indicate n per group in the figure legends.

3. In the introduction, please provide more detailed rational for focusing on the PRC, RSC, and PRL.

## Author Response

Thank you for reviewing our manuscript, “Touchscreen-based cognitive training alters functional connectivity patterns in aged but not young male rats.” We appreciated hearing from your expert reviewers. We were happy that reviewers felt “positively” about our manuscript and felt our study was “very well written” and “clearly presented.” We are also grateful that the reviewers spent time collaboratively examining our manuscript and offering thoughtful and well-reasoned suggestions. Below we have included our point-by-point reply to each reviewer question. We have also made changes in the manuscript document and highlighted them for ease of re-review. We feel these revisions have made the manuscript stronger and more impactful. We hope that you feel we have satisfactorily addressed each reviewer comment and that the manuscript is ready for publication.

1. ”...a limitations paragraph in the discussion should include consideration of the implications with only studying male subjects.”

Thank you for this important comment. We added a paragraph to the Discussion outlining the importance of studying both male and female subjects.

2. "Please also include paragraph subheadings to guide the discussion.”

Section headings have been added to the discussion.

3. "Effect sizes should be added for each significant effect in the cognitive training section.”

Effect sizes have now been added to all significant statistical analyses in the Results section.

4. "The control condition for the cognitive studies is a locomotor condition where the animals walk in a small circular environment to match the PAL condition. Locomotion and PAL was matched in time but did the authors directly compare the amount of locomotion by each animal (cumulative distance travelled)? The authors could add some additional analyses to describe the amount of activity during these sessions (e.g., number of laps or total distance travelled).”

The Walking Control task was designed to match the Working Memory (WM)/biconditional association task (BAT), which our lab has used in previous studies exploring functional connectivity changes following cognitive training in young and aged rats (Colon-Perez et al., 2019). Specifically, the Walking Control task was designed to match this task for time (30-45 minutes), number of trials (32 laps per day), and distance traveled. Specifically, the track itself had an outer diameter of 115 cm and an inner diameter of 88 cm, which gives the reviewers an idea of the total distance traversed by each rat each day (between 3680 and 2816 cm daily).

As mentioned by the reviewers, the PAL and activity-matched control tasks were matched for time. Given that trial completion took significantly less time in the PAL task compared to the WM/BAT and Walking control task, these conditions were not matched for trial number.

There is no easy way to quantify movement during PAL task completion. The operant chambers were not large enough to allow rodents to make “laps’ during testing (21.6 × 17.8 × 12.7 cm). Movement, outside of response to the touchscreen monitor, was not tracked with reasonable resolution. Therefore, it is difficult to measure physical effort expended during the PAL task.

We added the number of laps for the Walking Control task to the Methods section and noted the differences in the physical demands of these tasks in the Discussion.

5. "Figure 3. It is interesting that there is a completely different pattern of whole brain connectivity results for the animals testing in PAL vs. the control walking condition. The difference is especially striking at scan 1 prior to PAL training (when shaping has only been conducted), and the pattern of results is more similar after PAL training. It would interesting to compare measures across the two conditions throughout the results section and if appropriate emphasize some of these differences in the discussion section.”

We agree with the reviewers that our manuscript reveals interesting differences in baseline whole brain connectivity between the animals, particularly young, in the PAL versus the control Walking condition. This highlights an important difference in the cognitive load of the Cognitive Training condition compared to the Walking condition prior to scan 1.

Rats in both the Cognitive Training and Walking conditions were food restricted, requiring handling and regular weighing, prior to scanning. For rats that completed cognitive training, the first scan was acquired after all shaping procedures had been completed. Shaping procedures (Magazine, Any Touch, Must Touch, Must Initiate, and Punish Incorrect) involved introduction of the rat to the touchscreen chamber, training association of a liquid reward and interaction with the touchscreen, training specific association of a liquid reward and interaction with stimuli shown on the touchscreen, trial initiation, and punishment in response to an inappropriate interaction with the touchscreen. In contrast, rats in the Walking condition traversed a circular track for 30-45 minutes daily, 32 laps/day, for 11 days prior to the first scan. Although not as cognitively demanding as the PAL test phase, the PAL shaping and behavioral procedures completed before scan 1 are likely more cognitively demanding than the behavioral procedures completed before scan 1 by rats in the Walking condition.

This is likely why we see differences in functional connectivity metrics at scan 1 across training conditions, particularly in the young rats. Specifically, connectivity differences between food restricted young animals with no PAL shaping, and food restricted young animals with PAL shaping suggest that young brains also respond to cognitive training. Even more, this finding suggests that perhaps we would see similar changes in young rats relative to the aged rats in response to PAL training if we changed our scanning procedures, i.e., scanning before shaping procedures took place compared to afterward. These findings also make clear that the way the brain responds to cognitive training with the PAL task differs across young and aged animals.

Future research is needed to explore the modifiability of network architecture in the young brain; specifically, what types of cognitive factors and experiences the young brain responds to. In the meantime, the Walking control continues to act as a helpful control in determining differences between functional connectivity changes across scans occurring in response to cognitive training versus occurring in response to consistent physical activity.

We added comments throughout the Results section comparing functional connectivity metrics at scan 1 across ages and conditions and emphasized these differences in the Discussion.

6. "Figure 4C,D - A rationale should be presented as to why the top 20 highest degree nodes were selected for these analyses.”

Great question: Colon-Perez and colleagues (Colon-Perez et al., 2019) completed a similar study looking at differences in functional brain changes in response to cognitive training. Given observation of an increased representation of nodes with high strengths across scan sessions in aged rats, they examined the connectivity patterns of these nodes in the aged and young rats across scans. They did not find a similar increase in the connectivity of these nodes in the young rats across scans.

Similarly, we observed an increase in node strengths and degree across scan sessions in our aged rats. Given a lack of evidence that the PAL task is supported by unilateral brain structures, we decided to look at the 10 highest strength/degree nodes from each hemisphere. We observed an increase in connectivity between these nodes for aged rats in the Cognitive Training condition, but not young Training rats or rats in the Walking condition. Further, we looked at the modularity of these nodes, and found increased subnetwork modularity in aged rats following cognitive training and not in young rats or rats in the Walking control.

We added further rationale for these analyses to the Methods section.

7. "The limitations for the implications of these findings should be elaborated on in the discussion, especially since the animals are in a sedative state during the fMRI imaging process. There was only one sentence referencing previous studies of sedation during fMRI BOLD imaging in both human and rodent studies, however, this needs to be elaborated on. Did these studies look at resting state following a task? Particularly a learning and memory task? How are the results in this study confounded by the fact that animals had to go under sedation during the fMRI task? What are the implications of these confounds when we want to think of this work for translational purposes? Alternatively, these experiments could be redone without the use of anesthesia (Stenroos et al 2018). If that’s not possible, then a more elaborate discussion on the limitations and significant alterations on the effects of anesthesia on resting state functional connectivity should be discussed.”

We appreciate the reviewers for pointing out an important limitation of our study and a potential confound, particularly for future translational studies. Unlike human studies, rodent studies of resting-state functional connectivity are typically completed while a rat is in a mildly sedated state. Inhalation anesthetic agents, such as isoflurane, can cause vasodilation and suppress neural activity (Masamoto et al., 2007; Williams et al., 2010; Febo, 2011). However, they are also preferred over intravenous injectable anesthetic agents due to fast recovery, lower mortality, and ease of control of sedative levels in the blood. Although functional neuroimaging in rodents can be completed in awake, head-fixed animals (Febo, 2011; Stenroos et al., 2018), acclimation to head restraint can result in chronic stress. This is important to consider given age-related differences in hypothalamic-pituitary-adrenal (Hampstead et al.) axis function and the presence of HPA axis dysfunction to age-related diseases (Gupta & Morley, 2014).

BOLD activation patterns have been successfully measured with low levels of anesthetics in animals (Masamoto et al., 2007; Kannurpatti et al., 2008; Kim et al., 2010; Williams et al., 2010) and humans (Riehl et al., 2018). Longitudinal rsfMRI studies have also been completed in which functional connectivity changes were measured in sedated rodents after cognitive training with a learning and memory task (Ash et al., 2016; Nasrallah et al., 2016; Colon-Perez et al., 2019). Further, in the present study, isoflurane levels were kept low during functional imaging to limit dose-dependent vasodilation.

We added further information about sedative use in fMRI imaging to the Methods and Results section.

8. "For the figures, please show each subject represented on the bar graphs and indicate n per group in the figure legends.”

We added scatter points representing each subject to each graph, and added the total n per group to the figure legends.

9. "In the introduction, please provide more detailed rational for focusing on the PRC, RSC, and PRL.”

Thank you again for providing this comment. In the Abstract and Introduction, we clarified the importance of the medial temporal lobe (including the perirhinal cortex and hippocampus), in addition to the retrosplenial cortex and frontal cortical areas (prelimbic and infralimbic cortices), to associative learning and memory.

REFERENCES

Ash JA, Lu H, Taxier LR, Long JM, Yang Y, Stein EA, Rapp PR (2016) Functional connectivity with the retrosplenial cortex predicts cognitive aging in rats. Proceedings of the National Academy of Sciences of the United States of America 113:12286-12291.

Colon-Perez LM, Turner SM, Lubke KN, Pompilus M, Febo M, Burke SN (2019) Multiscale Imaging Reveals Aberrant Functional Connectome Organization and Elevated Dorsal Striatal. eNeuro 6.

Febo M (2011) Technical and conceptual considerations for performing and interpreting functional MRI studies in awake rats. Front Psychiatry 2:43.

Hampstead BM, Stringer AY, Stilla RF, Deshpande G, Hu X, Moore AB, Sathian K (2011) Activation and effective connectivity changes following explicit-memory training for face-name pairs in patients with mild cognitive impairment: a pilot study. Neurorehabil Neural Repair 25:210-222.

Kannurpatti SS, Biswal BB, Kim YR, Rosen BR (2008) Spatio-temporal characteristics of low-frequency BOLD signal fluctuations in isoflurane-anesthetized rat brain. NeuroImage 40:1738-1747.

Kim T, Masamoto K, Fukuda M, Vazquez A, Kim SG (2010) Frequency-dependent neural activity, CBF, and BOLD fMRI to somatosensory stimuli in isoflurane-anesthetized rats. NeuroImage 52:224-233.

Masamoto K, Kim T, Fukuda M, Wang P, Kim SG (2007) Relationship between neural, vascular, and BOLD signals in isoflurane-anesthetized rat somatosensory cortex. Cereb Cortex 17:942-950.

Nasrallah FA, Vinh To X, Chen D-Y, Routtenberg A, Chuang K-H (2016) Functional connectivity MRI tracks memory networks after maze learning in rodents. NeuroImage 15:196-202.

Riehl JR, Palanca BJ, Ching S (2018) High-energy brain dynamics during anesthesia-induced unconsciousness. Netw Neurosci 1:431-445.

Stenroos P, Paasonen J, Salo RA, Jokivarsi K, Shatillo A, Tanila H, Grohn O (2018) Awake Rat Brain Functional Magnetic Resonance Imaging Using Standard Radio Frequency Coils and a 3D Printed Restraint Kit. Front Neurosci 12:548.

Williams KA, Magnuson M, Majeed W, LaConte SM, Peltier SJ, Hu X, Keilholz SD (2010) Comparison of alpha-chloralose, medetomidine and isoflurane anesthesia for functional connectivity mapping in the rat. Magn Reson Imaging 28:995-1003.
